# Evolutionary Trade-Offs Underlie the Multi-faceted Virulence of *Staphylococcus aureus*

**DOI:** 10.1371/journal.pbio.1002229

**Published:** 2015-09-02

**Authors:** Maisem Laabei, Anne-Catrin Uhlemann, Franklin D. Lowy, Eloise D. Austin, Maho Yokoyama, Khadija Ouadi, Edward Feil, Harry A. Thorpe, Barnabas Williams, Mark Perkins, Sharon J. Peacock, Stephen R. Clarke, Janina Dordel, Matthew Holden, Antonina A. Votintseva, Rory Bowden, Derrick W. Crook, Bernadette C. Young, Daniel J. Wilson, Mario Recker, Ruth C. Massey

**Affiliations:** 1 Department of Biology and Biochemistry, University of Bath, Bath, United Kingdom; 2 Department of Medicine, Columbia University, New York, New York, United States of America; 3 Department of Medicine, University of Cambridge, Cambridge, United Kingdom; 4 School of Pharmacy and Pharmaceutical Sciences, Cardiff University, Cardiff, United Kingdom; 5 Pathogen Genomics, Wellcome Trust Sanger Institute, Wellcome Trust Genome Campus, Hinxton, Cambridge, United Kingdom; 6 School of Medicine, Medical & Biological Sciences, North Haugh, St Andrews, Fife, United Kingdom; 7 Nuffield Dept. of Medicine, University of Oxford, John Radcliffe Hospital, Oxford, United Kingdom; 8 Wellcome Trust Centre for Human Genetics, Oxford, United Kingdom; 9 Centre for Mathematics and the Environment, University of Exeter, Penryn Campus, Penryn, United Kingdom; Stanford University, UNITED STATES

## Abstract

Bacterial virulence is a multifaceted trait where the interactions between pathogen and host factors affect the severity and outcome of the infection. Toxin secretion is central to the biology of many bacterial pathogens and is widely accepted as playing a crucial role in disease pathology. To understand the relationship between toxicity and bacterial virulence in greater depth, we studied two sequenced collections of the major human pathogen *Staphylococcus aureus* and found an unexpected inverse correlation between bacterial toxicity and disease severity. By applying a functional genomics approach, we identified several novel toxicity-affecting loci responsible for the wide range in toxic phenotypes observed within these collections. To understand the apparent higher propensity of low toxicity isolates to cause bacteraemia, we performed several functional assays, and our findings suggest that within-host fitness differences between high- and low-toxicity isolates in human serum is a contributing factor. As invasive infections, such as bacteraemia, limit the opportunities for onward transmission, highly toxic strains could gain an additional between-host fitness advantage, potentially contributing to the maintenance of toxicity at the population level. Our results clearly demonstrate how evolutionary trade-offs between toxicity, relative fitness, and transmissibility are critical for understanding the multifaceted nature of bacterial virulence.

## Abstract 2

This study shows that, contrary to expectation, toxin secretion inversely correlates with disease severity for the major human pathogen *Staphylococcus aureus*.

## Author Summary

Global efforts to counter the growing problem of antibiotic resistance and develop alternative treatment strategies rely on a fuller understanding of when and why opportunistic pathogens cause disease. Recent advances in DNA sequencing technologies have opened up new opportunities to study infectious organisms, yet identifying the genetic variants that explain differences in disease remains challenging. Here we aimed to understand the complex relationship between toxicity—a known risk factor for disease in many bacteria—and infection severity for the major human pathogen *S*. *aureus*. Against expectations, we found that the bacteria that caused the most severe disease were the least toxic strains. We were able to determine the mutations responsible for the differences in toxicity and identified a number of novel toxicity-affecting genes. We further discovered that bacterial fitness in human serum could explain the unexpected association of low-toxicity isolates with severe, invasive disease. Invasive *S*. *aureus* infections are usually considered a dead end for these bacteria, as these infections are rarely transmitted to another person. Here we show using a simple mathematical model that this might in fact favour transmission of highly toxic bacteria on a population level and thus contribute to their global success. Our work therefore highlights the complexity of bacterial infection and should aid in devising new treatment and control strategies against this important pathogen.

## Introduction

The development of effective, long-term control strategies against microbial pathogens crucially relies on a thorough understanding of the many factors that contribute to the evolution and maintenance of enhanced virulence. Bacterial toxins are well established as playing a key role in virulence [1–4]. They release nutrients for bacterial growth and facilitate intra- and interhost transmission by destroying local tissue and subverting host immune processes [5–7]. This has led to the general presumption that elevated toxicity is positively associated with enhanced disease severity in bacterial infections [5,8,9]. However, the expression of toxins is readily switched off in vitro in response to the selection imposed by the energetically costly nature of their production [10,11]. Observational studies in *S*. *aureus* suggest that this can also occur in vivo, indicating that the relationship between toxicity and disease severity is more complex than initially appreciated [12–17].

*S*. *aureus* is a major human pathogen and a global healthcare issue. It is considered opportunistic as it asymptomatically colonises its host but can occasionally cause diseases that range in severity from relatively minor skin and soft tissue infections (SSTI) to life-threatening cases of pneumonia and bacteraemia [9]. Toxicity has been accepted as playing a key role in the success of lineages such as USA300 [18] and ST93 [19], in which it has been suggested to increase transmissibility. Toxicity is also widely accepted as playing a significant role in the virulence of *S*. *aureus*, where many studies comparing high- and low-toxicity isolates in animal models of sepsis show that highly toxic isolates cause more severe disease symptoms [20–24]. However, it has recently been shown that *S*. *aureus* isolates from humans with invasive diseases, such as bacteraemia and pneumonia, are often impaired in their ability to express toxins (often referred to as Agr dysfunction) [12,13]. A strength of the collections of isolates considered in these studies is that they represent all of the isolates presenting in a given geographical region over a defined period of time. Unfortunately, this type of sampling brings with it a limitation, as such collections consequently also contain a wide range of genetic backgrounds and antibiotic resistance profiles that might confound any potential associations between toxicity and disease.

In this study, we applied a robust functional genomics approach to two collections of *S*. *aureus* isolates, with significant depth and breadth, where we have controlled for genetic background by sampling within specific clinically important lineages, including USA300. Our approach not only allowed us to make observations that challenge our understanding of the role of toxicity in the establishment of severe, invasive disease but also to identify the genetic polymorphisms involved. By using a combination of functional assays, we further identified bacterial fitness in human serum as an important factor that could limit the ability of highly toxic isolates to cause bacteraemia and would explain the observed negative relationship between toxicity and disease severity. The power of genomics to study past events is clear, and here we demonstrate its potential to also help us understand fundamental aspects of bacterial pathogenicity and their role in invasive disease.

## Results

To determine the relationship between toxicity and disease for *S*. *aureus*, we performed a detailed investigation of two large collections of fully sequenced clinical isolates representing significant breadth and depth of sampling (S1 Table). The first collection (ST15, PVL-, MSSA) was isolated from a single patient who progressed from asymptomatic carriage to bacteraemia over a 15-mo period [25]. The second, a collection of 134 clinical isolates, all corresponding to a single clone (USA300, ST8, PVL+, SCCmec type IV MRSA) and isolated from the nose or skin of healthy volunteers (carriage), from SSTI or from bacteraemic patients [26].

### Reduced Toxicity Associates with Invasive Disease

**Fig 1:**
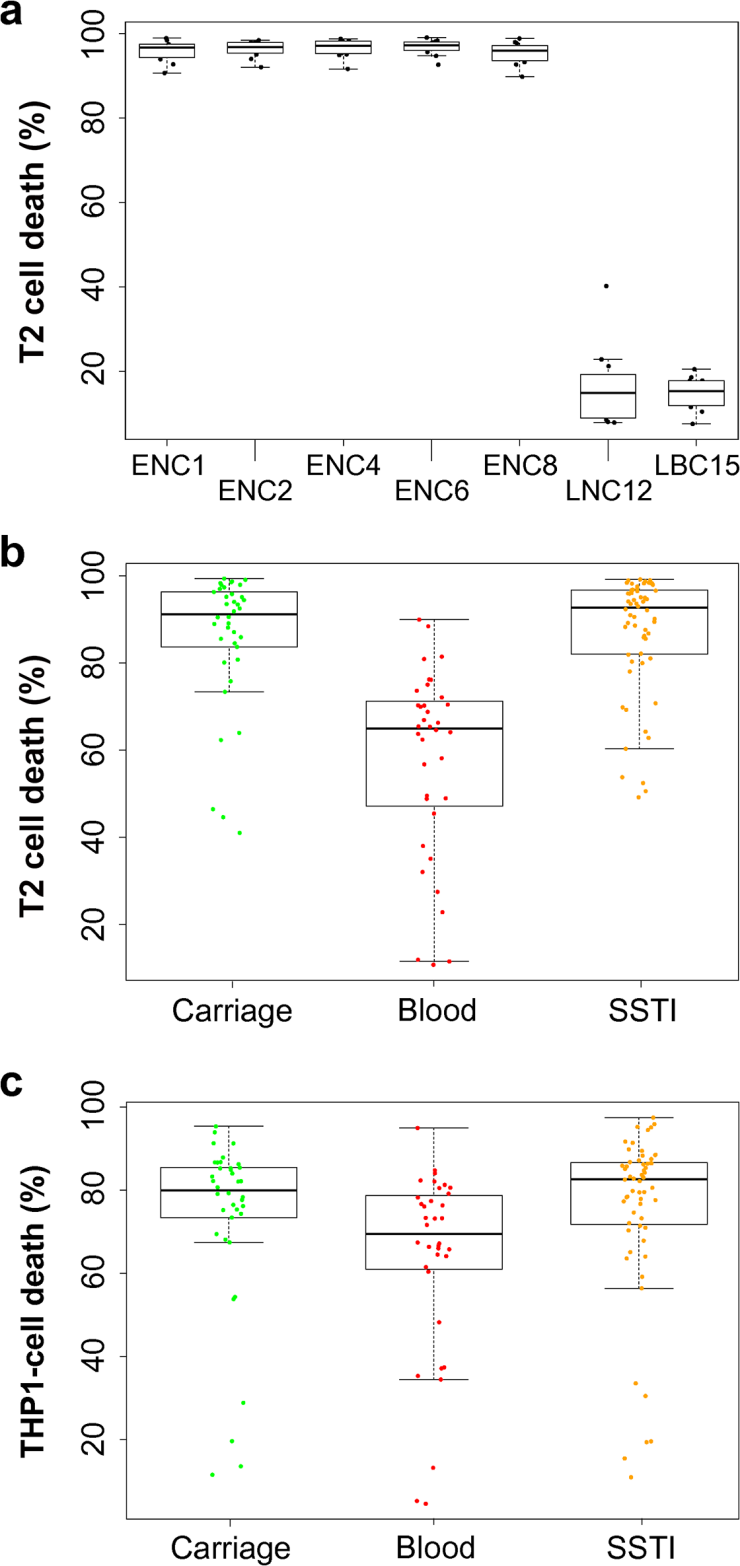
Low levels of toxicity are associated with invasive *S*. *aureus* infections across two collections of clinical isolates. **a:** Individual isolates from seven time points were assayed for toxicity and their genome sequenced. Early nose cultures (ENCs) from months 1, 2, 4, 6, and 8 were all highly toxic. Late nose culture (LNC) isolates at month 12 and bloodstream isolates following bacteraemia (late blood culture, LBC) were all significantly less toxic than the ENC isolates. **b** and **c:** The toxicity of 134 USA300 isolates were assayed, and those from bacteraemia were on average significantly less toxic (measured as percent T2 and THP1 cell death (b and c, respectively)) than from carriage or SSTI *(p <* 0.01 ANOVA). The medians are presented as horizontal bars, with the boxes and whiskers showing the 1st and 3rd quartile and interquartile ranges. To access this data, see S1 Data.

The single-patient collection included serial asymptomatic nasal carriage isolates over a 12-mo period, as well as bloodstream isolates after bacteraemia had developed at month 15 [25]. At each time point, 12 individual colonies were isolated from the primary plates for each swab. These isolates all belong to the ST15 lineage and contain genes for 12 of the 13 known cytolytic toxins secreted by *S*. *aureus* (i.e., alpha, beta, gamma, delta, LukAB, LukED, PSMα1, α2, α3, α4, β1, β2, but not LukSF [a.k.a. Panton-Valentine-leukocidin or PVL]). To quantify the gross cytolytic activity of each isolate, we used an immortalised T/B hybridoma cell line (T2), which is susceptible to 10 of the 12 cytolytic toxins present in this collection (not LukAB or LukED) (S1 Fig). Despite some sequence variability across the 12 isolates from each time point and between the different time points [25], no diversity in toxicity was observed for the early nasal culture isolates (Fig 1A). At month 12, however, there was a significant drop in toxicity for all 12 nasal carriage colonies, shortly after which the study participant developed bacteraemia (month 15). The bacteria isolated from the patient’s bloodstream also showed significantly reduced toxicity compared to those from the earlier time points (Fig 1A), demonstrating an apparent inverse correlation between toxicity and disease, albeit in a sample size of only one patient.

To ensure this effect was not a consequence of the lack of sensitivity of the T2 cell line to LukAB and ED, we quantified the ability of a subset of six high- and six low-toxicity isolates (as determined using the T2 cells) to lyse freshly harvested human neutrophils, which are sensitive to LukAB and LukED. We found that the isolates that were unable to lyse the T2 cells were also unable to lyse the neutrophils (S1 Fig), suggesting that LukAB and LukED were not expressed by these low-toxicity isolates, and that the T2 cell line provides a robust measure of gross cytolytic activity for this collection of isolates.

Having demonstrated a negative correlation between toxicity and disease for a single patient, we sought to extend this to multiple patients by focussing on a collection of 134 USA300 MRSA isolates. As the USA300 lineage is known to contain the genes encoding the LukSF (i.e., PVL) toxin we included a human monocytic cell line, THP-1, which is sensitive to the action of this toxin [27]. In this set of isolates, we again found that the bacteraemic isolates were significantly less toxic than either the carriage or the SSTI groups (Fig 1B and 1C, ANOVA: *p* < 0.01 for both T2 cells and THP-1 cells). With these two collections of isolates, representing both broad sampling across patients and deep sampling within a patient, we demonstrate a significant negative association between toxicity and disease severity.

### Polymorphisms in a Range of Loci Affect the Toxicity of *S*. *aureus*

**Fig 2:**
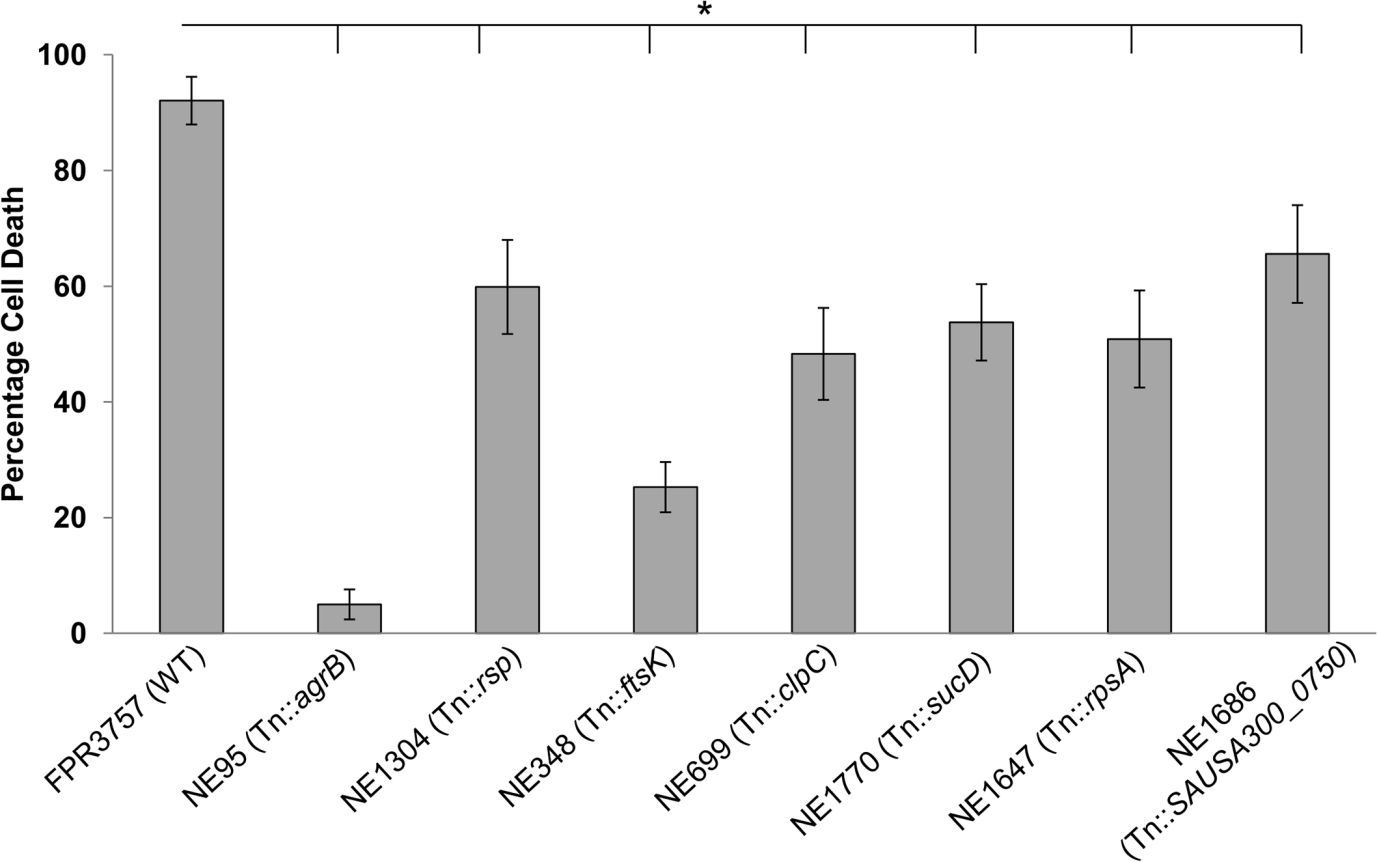
The identification of six novel toxicity-affecting loci. Transposon mutagenesis was used to functionally verify the role of loci associated with toxicity by genome-wide association study (GWAS; PLINK). Mean of six replicates are presented, error bars represent the 95% confidence intervals. The mutation in *agrB* is provided as a negative control. To access this data, see S2 Data.

The single-patient collection contained only a small number of genetic differences between the isolates, and as such we were readily able to identify the genetic basis of the change in toxicity. We found that the late nasal carriage and blood culture isolates had a premature stop-codon in an *araC*-like transcriptional regulator gene *rsp*, which encodes a protein that has previously been shown to regulate biofilm formation by this pathogen [28]. Using a transposon insertion in the *rsp* gene (NE1304) from the Nebraska library [29], we found a significant reduction in toxicity compared to the wild-type strain (Fig 2), thus verifying this gene’s toxicity-regulating function. Interestingly, mutating the *rsp* gene with a transposon in the USA300 (ST8) background (Fig 2) did not have as significant an effect on toxicity as the stop-codon in the ST15 background (Fig 1A), presumably as a consequence of differences in the genetic background of these bacterial clones.

To identify the genetic polymorphisms responsible for the changes in toxicity of the USA300 isolates, we adopted a functional genomics approach using genome-wide association studies (GWAS) to identify candidate polymorphisms associated with toxicity (S2 Table). As GWAS is prone to high false positive rates, we sought to verify the effect of the associated loci using transposon insertions in each locus that was available from the Nebraska library (the full list of significantly associated polymorphisms can be found in S2 Table). We identified five novel toxicity-affecting loci (Fig 2): *ftsK*, *clpC*, *sucD*, *rpsA*, plus a hypothetical gene with no known activity or homology to other proteins (SAUSA300_0750). Based on amino acid homology, the FtsK protein is believed to be a DNA translocase, where polymorphisms have also been associated with changes in the toxicity of another globally successful MRSA clone, ST239 [30]. FtsK also shares significant structural similarities to transporters, suggesting that this protein may be directly involved in the secretion of toxins from the bacterial cell, rather than DNA translocation. The ClpC protein is a chaperone and has been shown previously to affect the expression of a large number of proteins, including several regulators of toxin expression [31]. The role of the proteins encoded by *sucD* (succinyl-CoA synthetase subunit alpha) and *rpsA* (30S ribosomal protein S1) in toxin expression is less clear, although any changes to the metabolism of a cell are likely to have significant downstream effects on gene expression [32]. Further work is underway to elucidate the molecular detail of how these proteins affect toxicity.

### Explaining the Association between Toxicity and Infection Severity

#### A) Underlying health of patient

**Fig 3:**
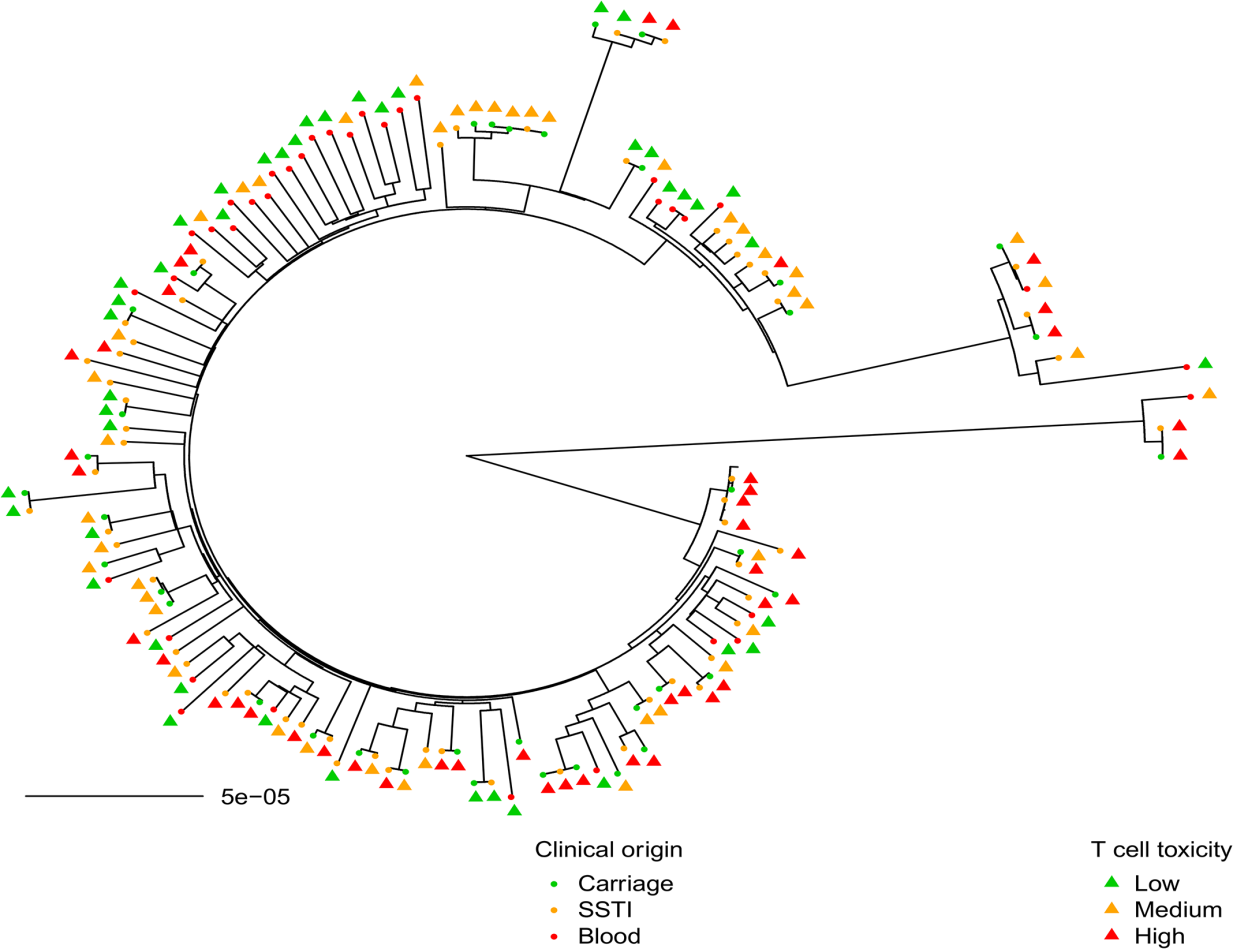
Toxicity levels and disease origin distributed across the USA300 collection. The clinical origin (carriage = C, SSTI = S, or blood = B) and the toxicity (low = green, medium = orange or high = red) of 134 USA300 isolates are mapped onto a maximum likelihood tree based on their whole genome sequence.

It is possible that the less toxic isolates are more often associated with bacteraemia because of the presence of comorbidities associated with an impaired immune system that might result in failure to control less toxic organisms. Although we cannot fully discount this, when we examined the relatedness of these isolates by mapping the isolate source and level of toxicity onto a maximum likelihood tree based on their genome sequence (Fig 3), despite some clustering of the bacteraemic isolates, they are distributed across the tree and are often closely related to highly toxic carriage or SSTI isolates. This suggests that a susceptible patient would be just as or more likely to be exposed to a highly toxic as to a low toxicity isolate. A retrospective chart review (summarised in S3 Table) also found no associations between the presence of suspected portals of entry, types of suspected portals of entry, or length of bacteraemia with the toxicity of the isolates. While the health of the patient is undoubtedly a factor in their susceptibility to the development of bacteraemia, where the damage-response framework is clearly implicated [33], it does not explain why patients are more likely to develop invasive diseases with the less toxic isolates.

#### B) Cell-invasive capacity

**Fig 4:**
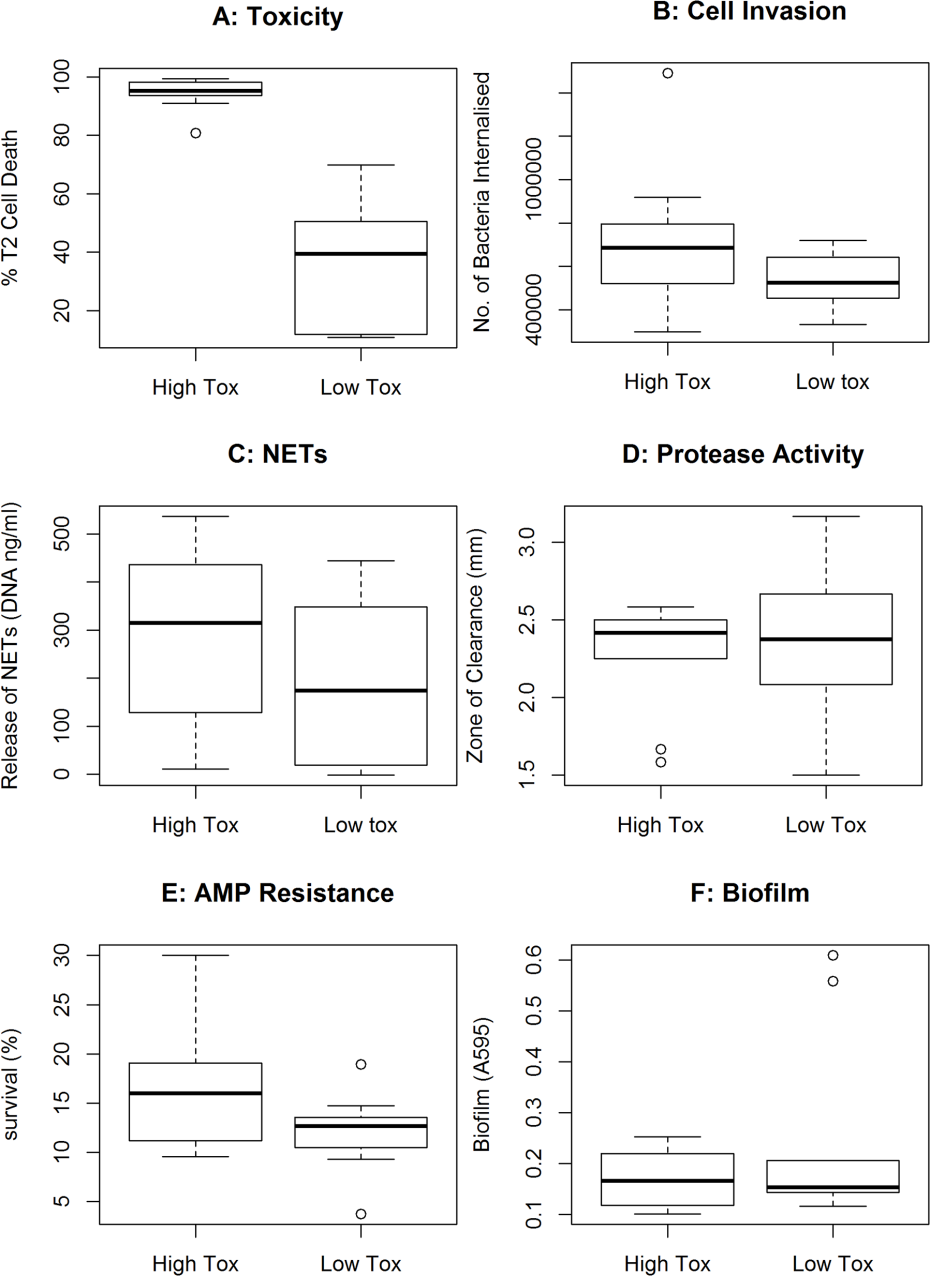
Explaining the association between toxicity and infection severity. A range of virulence assays were performed on a subset of 10 high- and 10 low-toxicity isolates from the USA300 collection to identify the biological feature that explains the observed inverse correlation between toxicity and disease severity. (A) The toxicity of the subset of isolates is provided for comparison. (B) The ability of the bacteria to invade EA.hy926 endothelial cells. (C) The ability of the bacteria to induce the release of neutrophil extracellular traps (NETs). (D) The release of proteases by the bacteria. (E) Resistance of the bacteria to human neutrophil defensin-1. (F) The ability of the bacteria to form biofilms. The *p*-value for the comparison between the high and low toxicity isolates was greater than 0.05 for all traits with the exception of toxicity. The medians are presented as horizontal bars, with the boxes and whiskers showing the 1st and 3rd quartile and interquartile ranges. To access this data see S3 Data.

The regulation of toxin secretion by *S*. *aureus* has been well studied under laboratory conditions [34,35], where they are known to phenotypically switch between their ability to attach to and invade human cells and their ability to secrete toxins. We therefore hypothesised that the more toxic isolates might be impaired in their ability to invade human cells, causing them to be less able to gain entry to the bloodstream. To test this hypothesis, we quantified the ability of 10 high- and 10 low-toxicity isolates to invade a human-derived endothelial cells line (EA.hy926) and found no differences in cell-invasive capacity between high- and low-toxicity isolates (Fig 4B).

#### C) The release of neutrophil extracellular traps (NETs)

Another feature of the ability of *S*. *aureus* to cause disease is their immune evasive capacity. Neutrophils are of particular importance in immunity to *S*. *aureus* infection, and of the many roles they play, their ability to trap bacteria in NETs has been shown to be triggered by cytolytic toxins [36]. We therefore hypothesised that neutrophils in the bloodstream might be more efficient at trapping the more toxic isolates, impeding their ability to establish an infection in the bloodstream. However, when we compared the ability of 10 high- and 10 low-toxicity isolates to induce neutrophil net formation, while we observed significant differences between individual isolates, this was unrelated to their toxicity (Fig 4C).

#### D) Protease activity

As the secretion of proteases by *S*. *aureus* also causes significant tissue damage, we hypothesised that the more toxic isolates might be less proteolytic, and this could impede their ability to cause bacteraemia. To test this, we assayed the protease activity of 10 high- and 10 low-toxicity isolates; however, no significant difference in protease activity between these collections of isolates was observed (Fig 4D)

#### E) Antimicrobial peptide resistance

A fundamental aspect of host innate immunity that provides protection from bacterial infection is the secretion of antimicrobial peptides. We hypothesised that the more toxic isolates may be less resistant to the effect of these, which would impede their ability to cause bacteraemia. To test this, we measured the percentage survival of a subset of 10 high- and 10 low-toxicity isolates following incubation with 5 μg/mL human neutrophil defensin-1 (hNP-1), but no significant difference between these collections of isolates was observed (Fig 4E)

#### F) Biofilm formation

As biofilm formation by *S*. *aureus* is believed to contribute to their ability to colonise both biotic and abiotic surfaces, we hypothesised that the high toxicity isolates may be less able to form biofilm, impeding their ability to cause bacteraemia. To test this, we quantified the ability of 10 high- and 10 low-toxicity isolates to form biofilms; however, no difference between the high and low toxicity isolates was observed (Fig 4F).

#### G) Relative fitness in human serum

**Fig 5:**
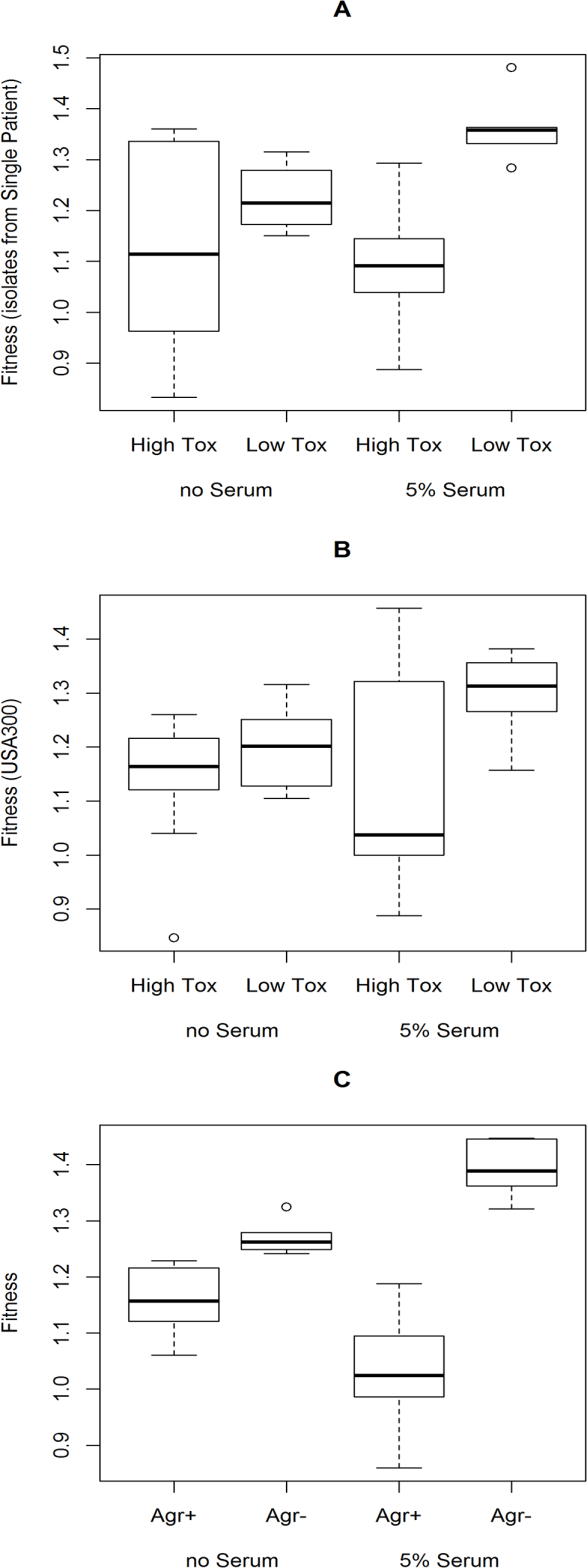
The highly toxic isolates are less fit than the low-toxicity isolates in the presence of human serum. We quantified the relative fitness of six high- and six low-toxicity isolates from the single patient collection (A), 10 high- and 10 low-toxicity isolates from the USA300 collection (B) and an Agr wild type and mutant isogenic pair (C) in BHI with and without 5% (vol/vol) human serum. The medians are presented as horizontal bars, with the boxes and whiskers showing the 1st and 3rd quartile and interquartile ranges. *p*-values are indicated in the text. To access this data, see S4 Data.

The bloodstream is a highly protected niche. With almost all elements of host immunity present, it acts as a severe bottleneck for the bacteria, as evidenced by the lack of genetic diversity amongst the isolates from bacteraemic patients (we found no genetic diversity amongst them, despite the 12 isolates having been sampled at two time points and collected in three bottles). Serum contains many antimicrobial peptides and has been shown in two previous studies to have the effect of both increasing the expression of toxins [37,38] and neutralising their activity simultaneously [39]. As toxin expression is widely accepted as being an energetically costly activity, we hypothesised that the fitness costs associated with the up-regulation of toxin expression in blood could be sufficient to reduce the probability of the highly toxic isolates from getting through the bottleneck and establishing a blood stream infection. We therefore compared the growth dynamics and relative fitness of six high- and six low-toxicity isolates from the single-patient collection and 10 high- and 10 low-toxicity isolates from the USA300 collection in brain–heart infusion broth (BHI) and in BHI supplemented with 5% human serum. Although 5% serum had significant antibacterial activity on these isolates (S2 Fig and S6 Data), we observed no difference in growth dynamics of the high and low toxicity isolates. However, when we measured the relative fitness of the isolates, which is more sensitive to growth defects, we found that in BHI the high-toxicity isolates from both collections were slightly less fit than the low-toxicity isolates, although not statistically significant (*p* = 0.19 and 0.28 respectively, Fig 5A and 5B). However, when serum was added, which has the effect of increasing toxin expression further, the difference in relative fitness between the high- and low-toxicity isolates was significantly increased for both collections (*p* = 0.003 and 0.02 respectively, Fig 5A and 5B).

To support our hypothesis that the effect on fitness is due to levels of toxin expression, we ensured that the low-toxicity isolates all had SNPs in a wide range of loci (including *rsp*, *ftsK*, *clpC*, *sucD*, *rpsA*, and SAUSA300_0750), and further included an Agr wild type and mutant isogenic pair (Fig 5A–5C). Regardless of the locus affected, once toxicity was lowered, relative fitness was increased upon exposure to serum.

### The Observed Association between Low Toxicity and Bacteraemia May Contribute to the Maintenance of High Toxicity at a Population Level

With mortality rates as high as 20% for *S*. *aureus* bacteraemia [40], understanding how these types of infections develop is of significant clinical importance. However, as the bacteria rarely transmit beyond this point, they can be effectively thought of as transmission dead ends. We therefore sought to explore the possible evolutionary effect of the observed association of low-toxicity isolates with invasive infections using a simple mathematical model (see Methods). We considered two competing strains of *S*. *aureus* that differed in their level of toxicity, where higher toxicity was assumed to be positively correlated with transmissibility and progression from carriage to SSTI, based on the fact that we rarely find low-toxicity isolates amongst our carriage or SSTI populations, and that low-toxicity mutants have been rarely found to transmit amongst healthy populations [17]. But higher toxicity also resulted in faster treatment. With this, we examined what effect the strains’ relative propensity to cause bacteraemia (σ) had on their competitive fitness.

**Fig 6:**
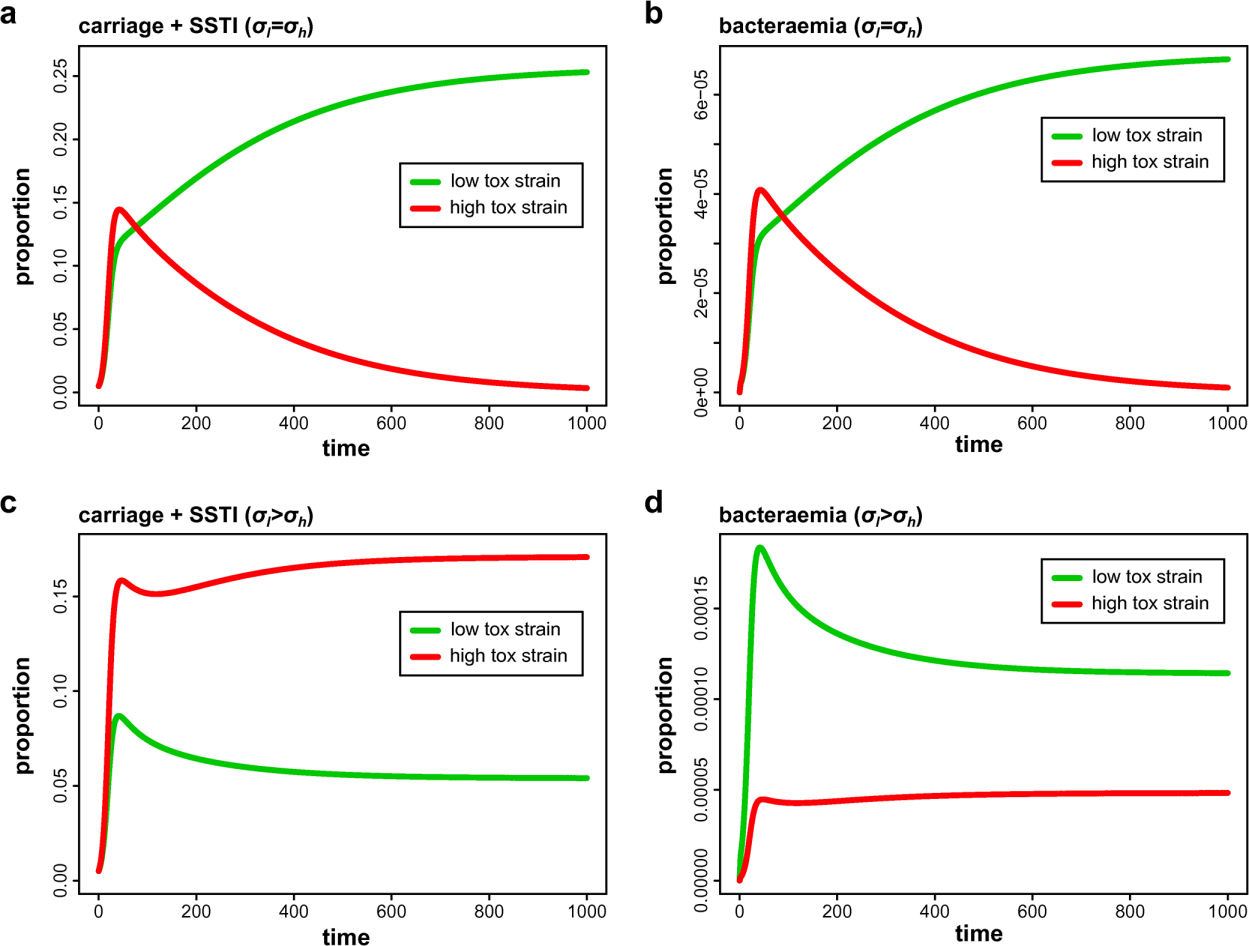
Evolution towards increased levels of virulence. Two competing MRSA strains with different levels of toxicity are considered, where higher toxicity is assumed to increase the transition rate from carriage to infection (SSTI) but also increases treatment-induced clearance of infections. At baseline, we assume that both strains have equal propensities to cause severe bacteraemia (*σ*_*l*_ = *σ*_*h*_), which causes the more toxic strain (red) to be outcompeted due to its faster, virulence-induced clearance rates and leads to the dominance of the less toxic strain (green) both at carriage + SSTI (**a**) and at bacteraemic stage (**b**). By considering a negative correlation between toxicity and bacteraemia (*σ*_*l*_ *> σ*_*h*_), the more toxic strain gains a competitive advantage, leading to higher frequencies at carriage and SSTI stages (**c**). However, the higher propensity of the less toxic strain, in this case maintained in the population through mutational down-regulation from highly toxic strains, maintains its high frequency at the bacteraemic stage (**d**). See Methods for parameter values.

Assuming no differences (*σ*_*l*_ = *σ*_*h*_, Fig 6A and 6B and S3 Fig), we find that the higher clearance rate of the more toxic strain offsets its transmission advantage, leading to its exclusion and dominance of the less toxic strain (shown separately for carriage and SSTI and bacteraemia in Fig 6A and 6B, respectively). In contrast, considering the clinically observed negative association of toxicity with bacteraemia (*σ*_*l*_ *> σ*_*h*_) results in the more toxic strain gaining a competitive advantage at the population level (carriage and SSTI, Fig 6C, S3 Fig and S4 Fig), whereas the less toxic strain maintains its elevated frequency during bacteraemia (Fig 6D, S3 Fig and S4 Fig), in line with clinical observations. This suggests that the reduced opportunity for transmission due to bacteraemia could partly compensate for the toxicity-driven trade-off between transmissibility and treatment rates and thus contribute to the maintenance and circulation of highly toxic strains within the population.

## Discussion

For microbial pathogens, many factors contribute to their success, but for an opportunistic pathogen that can either reside asymptomatically or cause symptomatic infections ranging from superficial to life-threatening invasive disease, the definition of success becomes increasingly complex. The damage-response framework outlines the necessary holistic approach we need to take when considering this, where both the level of virulence expressed by the pathogen and the response of the host is critical to the clinical outcome [33]. For *S*. *aureus*, the vast majority of infections resolve without clinical intervention, and severe infections (e.g., bacteraemia or pneumonia) are generally limited to those with compromised health [9]. With many genes encoding cytolytic toxins, and highly toxic clones disseminating worldwide, it is understandable that toxicity and virulence have long been considered directly linked and key to its success. However, here we show this relationship to be quite complex.

The boom in genomic sequence data for microbial pathogens has allowed us to study past genetic events in great detail, tracing epidemics and studying how bacterial genomes evolve [25,41–44]. It is, however, only recently that we have been able to successfully use such genomic data to study the behaviour of pathogens and use functional genomics approaches to understand why and how specific traits evolve [30,45–49]. By studying two large collections of isolates, we demonstrate that bacteraemic isolates are significantly less toxic than those isolated from carriage or from SSTIs. With the genome sequence of each isolate available to us, we were able to identify the polymorphisms responsible for the observed changes in toxicity and in doing so have identified six novel toxicity-affecting loci for this pathogen. A molecular dissection of each locus is currently underway to determine how they affect this trait, but this work clearly demonstrates the power of functional genomics for studying bacteria.

To understand why low toxicity isolates have a higher propensity to cause bacteraemia, we developed and tested several hypotheses. While the health of a patient is a feature in their susceptibility to bacteraemia, we were unable to find evidence to suggest that this would increase their propensity to develop bacteraemia with a low rather than a highly toxic isolate. Furthermore, we found no evidence suggesting that either cell-invasiveness, NET formation, protease activity, antimicrobial peptide resistance, or biofilm formation play a role. Instead, we found that the presence of serum, which simultaneously increases toxin expression while neutralising their activity, reduces the relative fitness of the highly toxic isolates. Given the extreme bottleneck that the establishment of a bloodstream infection represents to bacteria, we believe this explains our finding that low-toxicity isolates are a more common cause of this type of invasive disease. By use of a mathematical model, we also showed that this increased propensity for low-toxicity isolates to cause bacteraemia, alongside the dead end nature of such infections, could potentially contribute to the maintenance of high levels of toxicity at a population level, as evidenced by the global prevalence of highly toxic clones, such as USA300 and ST93.

To understand the relative efficiency of GWAS to identify novel toxicity-affecting loci, we need to compare it to a similar experiment that used a random approach. Fey et al., who created the transposon library used here, screened their 1952 Tn mutants for haemolytic activity using rabbit blood agar plates and identified 71 mutants with a change in this phenotype [29]. Compare this hit rate of 3.6% with ours of 6.7% and the relative efficiency of this GWAS approach is apparent. This efficiency is, however, significantly affected by the size and relatedness of the isolates within the collection; as in a previous GWAS study, we used a more closely related collection of isolates and had a hit rate of 30% [30]. Such issues need to be considered when designing GWAS experiments for bacteria.

This work highlights a clear limitation to existing animal models of infection for pathogens like *S*. *aureus*. Studies published by us and many other groups have shown that in sepsis models, highly toxic isolates cause a more severe infection in mice than less toxic isolates [20–24], which would seem contrary to our findings here in humans. However, a major difference between these two systems needs to be considered. For humans, studies have demonstrated that bacteraemia represents an extreme bottleneck, with only a small number of cells being sufficient to cause disease [25,50]. For mice, on the other hand, 10^7^–10^8^ colony-forming units need to be injected directly into the tail vein to get reproducible infections, which overrides the rigours bacteria need to go through to cause bacteraemia naturally in human. Furthermore, subtle differences in relative fitness, which we suggest contribute to our clinical observations, would not be evident in a mouse overwhelmed with the introduction of so many bacteria cells directly into its bloodstream. We would therefore urge caution when interpreting the results from such experiments in relation to human disease.

It is interesting to note that no difference in toxicity was observed between the carriage isolates and those causing SSTIs, and that, on average, both groups were highly toxic. Many animal models have been used to demonstrate that the size of a cutaneous lesion is greatly affected by the levels of toxins secreted by the infecting organism [5]. While we have no clinical data to compare toxicity levels and lesion size for these isolates, that we rarely find low-toxicity isolates causing SSTIs supports the findings from animal models that toxicity correlates with the ability to cause such lesions. It is also intriguing to consider what selective forces maintain such a high level of toxicity at a population level. For symptomatic transmission, the toxin-induced production of highly transmissible pus has obvious benefits. However for asymptomatic transmission, this is less obvious. Low toxicity isolates can survive in the nose, as indicated by the single patient collection where the low toxicity isolates survived there for 3 mo before causing bacteraemia. However, perhaps over a longer term, the ability of highly toxic bacteria to resist the effects of nasal-associated immunity by killing host immune cells, and the use of some toxins (e.g., the phenol soluble modulins, PSMs) to interfere with competing genera of bacteria, is key to its maintained selection.

For *S*. *aureus*, an opportunistic pathogen, it is clear that virulence is multifaceted. On the one hand, the prevalence of highly toxic clones globally and the role of specific toxins in causing highly transmissible pus-filled SSTI lesions suggests that toxins offer a selective advantage. On the other hand, it appears that offsetting toxicity for short-term enhanced fitness is associated with increased virulence, which may paradoxically select for the maintenance of higher levels of toxicity at a population level. Although at a superficial level these seem contradictory, it is clear that they are critical aspects of this pathogen’s success. With the movement of genome sequencing into routine clinical practice and the drive towards personalised medicine, we need to define these complex interactions and bring the biology of the pathogen into greater consideration in clinical settings.

## Materials and Methods

### Ethics Statement

Whole blood was obtained from healthy volunteers; ethical permission for all donations was obtained from local research ethics committee (School of Pharmacy & Pharmaceutical Sciences Ethics Committee) and all participants gave written informed consent.

### Bacterial Strains and Growth Conditions

A list of the bacterial strains used can be found in S1 Table. For the toxicity assays, the *S*. *aureus* isolates were grown overnight in 5 mL of Tryptic-Soy Broth (TSB) in a 30 mL glass tube. This overnight culture was used to inoculate the toxicity-assay cultures at 1:1,000 dilution in fresh TSB and then grown for 18 h at 37°C in air with shaking (180 rpm). For transposon mutants, erythromycin (5 μg/mL) was included in the growth medium. The toxin-containing supernatant for each isolate was harvested by centrifugation.

### T2 and THP-1 Toxicity Assay

Immortalised human T2 cells and monocyte macrophage THP-1 cell lines were used as described previously [51]. The THP-1 cell line was included for the USA300 collection as it is susceptible to the PVL [27]. Briefly, both cell lines were grown in individual 30 mL suspensions of RPMI-1640, supplemented with 10% heat-inactivated fetal bovine serum (FBS), 1 μM L-glutamine, 200 units/mL penicillin, and 0.1 mg/mL streptomycin at 37°C in a humidified incubator with 5% CO_2_. Cells were routinely viewed microscopically every 48–60 h and harvested by centrifugation at 1,000 rpm for 10 min at room temperature and resuspended to a final density of 1–1.2 x 10^6^ cells/mL in tissue-grade phosphate buffered saline. This procedure typically yielded >95% viability of cells as determined by trypan blue exclusion and easyCyte flow cytometry. To monitor *S*. *aureus* toxicity, 20 μL of cells were incubated with 20 μL of bacterial supernatant and incubated for 12 min at 37°C. For the USA300 strains, supernatants were diluted to 30% of the original volume in TSB as these isolates were considerably more toxic than the single-patient isolates. Cell death was quantified using easyCyte flow cytometry using the Guava viability stain according to manufacturer’s instructions. Experiments were done in triplicate, and error bars indicate the average ± the 95% confidence interval of multiple independent experiments.

### GWAS

The identification of genetic variation in all the clinical isolates studied has previously been described [25,26] with the exception of the 36 bacteraemic USA300 isolates. These were sequenced in an identical manner to the others; namely, genomic DNA was extracted using the QIAamp DNA Mini Kit (Qiagen), and unique index-tagged libraries were generated. Whole-genome sequencing was carried out using the Illumina HiSeq2000 with 100-base paired-end reads. Paired-end reads were mapped against the core chromosome of the ST8 USA300 reference genome sequence FPR3757 (accession NC_02952) [52]. SNPs and indels were identified as described previously [53]. ENA accession numbers are listed in S1 Table. We conducted a quantitative association study on a set of 134 USA300 isolates to identify SNPs that were significantly associated with toxicity, using the PLINK software package (http://pngu.mgh.harvard.edu/purcell/plink/) [54]. These and a description of the loci are listed in S2 Table.

### Maximum Likelihood Tree

This was estimated using PhyML with an HKY85 substitution model, empirical nucleotide usage, no rate heterogeneity, and no invariant sites. The percentage toxicity range was divided by three, where the most toxic isolates were labelled red, the midtoxicity isolates labelled orange, and the least toxic isolates labelled green.

### Endothelial Invasion Assay

Bacterial invasion of EA. Hy926 endothelial cells were performed as described previously with minor modifications [55]. Endothelial cells were cultured in Dulbecco’s modified Eagles’ medium (DMEM) supplemented with 10% FBS and 2 mM L-glutamine at 37°C in a humidified incubator with 5% CO_2_. Cells were liberated using trypsin-EDTA solution, resuspended in culture medium, and aliquoted into 24-well tissue culture tissue plates and grown to >95% confluence. Cells were washed twice in tissue-grade PBS, and 450 μL of fresh DMEM was added. 50 μL of washed *S*. *aureus* (1 x 10^7^ CFU/mL) was added to the wells and incubated for 1 h at 37°C. Following incubation, the medium was aspirated and wells gently washed once in PBS and replaced with DMEM supplemented with 200 μg/mL gentamicin and incubated at 37°C for a further 60 min. Cells were subsequently lysed by the addition of 500 μL of Triton X-100, and bacterial CFU were enumerated by serial dilution of endothelial cell lysates and plating onto TSA plates and incubated at 37°C overnight. Experiments were performed in duplicate three times, and the error represents the 95% confidence interval.

### Formation of Neutrophil Extracellular Traps and Neutrophil Lysis Assay

Whole blood was obtained from healthy volunteers; ethical permission for all donations was obtained from a local research ethics committee (School of Pharmacy & Pharmaceutical Sciences Ethics Committee), and all participants gave written informed consent. Human neutrophils were isolated as previously described [56]. Cell-free supernatants from bacterial culture were diluted to 30% in warm Krebs buffer and then diluted 1:1 with prewarmed neutrophils (10^6^ neutrophils/mL in Krebs) and incubated at 37°C for 12 min. Cells were pelleted, and NET formation was quantified by measuring DNA content in the supernatant with Sytox Green and a DNA standard curve. Any signal from the bacterial culture was measured and subtracted from these values. For the neutrophil lysis assay, 20 μL of purified neutrophils was incubated with 20 μL of 10% bacterial supernatant for 15 min, and cell viability was assayed using Guava viability reagent and Guava flow cytometry.

### Protease Assay

A modified tryptic soy agar medium was made with 10% skim milk. 50 μL of bacterial supernatant harvested from overnight cultivation was inoculated into 1 cm diameter wells perforated into the agar medium and incubated for 18 h at 37°C. The digested substrate, as a result of proteolytic activity, was observed as clear areas surrounding the wells, was measured. Protease assay were done in duplicate, three times, and error represents the 95% confidence interval.

### Antimicrobial Peptide Resistance

Purified human neutrophil defensin-1 (hNP-1) was purchased from AnaSpec Incorporated (California, USA). The hNP-1 susceptibility assay was performed in 1% BHI with the addition of 10 mM potassium phosphate buffer as described previously [57]. A final inoculum of 10^5^ CFU, with a peptide concentration of 5 μg/mL, was employed and incubated for 2 h at 37°C. Final bacterial concentration was evaluated by serial plating onto TSA plates and data represented as mean (± SD) percent survival CFU/mL.

### Biofilm Formation

Semiquantitative measurements of biofilm formation on 96-well polystyrene plates was determined based on the method of Ziebuhr et al [58]. Overnight bacteria grown in TSB were diluted 1:40 into 100 μL TSB containing 1% glucose and grown for 24 h at 37°C. Following 24-h growth, plates were washed vigorously five times in PBS, dried and stained with 150 μL of 1% crystal violet for 30 min at room temperature. Following five washes of PBS, wells were resuspended in 200 μL of 7% acetic acid, and optical density at 595 nm was recorded using a plate reader.

### Relative Fitness Assay

*S*. *aureus* isolates were grown overnight in BHI broth to an OD_600_ of 2.0 to ensure that all cells are in a similar physiological state at the start of the experiment. Competitions were established in TSB with and without 5% (vol/vol) freshly drawn human serum. The competition medium was inoculated with 10^4^ CFU/mL of the marker strain (MSSA466, which is tetracycline resistant) and 10^3^ CFU/mL of the test strain. Initial cell numbers were confirmed by plating. The bacteria were competed at 37°C in a shaking incubator (180 rpm) for 24 h. Final cell numbers were enumerated by serial dilutions on TSA plates (total cell count) and TSA plates containing 2 μg/mL tetracycline (marker strain count). The fitness of a strain was defined as a measure of the reproductive success of the population, which can be expressed as the natural logarithm of the ratio of the final and initial cell densities of the culture [59]. Each clinical strain was assayed once as each was considered a biological replicate indicative of its group.

$$\text{relative}\;(\text{Darwinian})\;\text{fitness}=\frac{\text{ln}(A(1)/A(0))}{\text{ln}(M(1)/M(0))}$$

where: *A*(0), estimated density of test strain at time 0; *M*(0), estimated density of marker strain at time 0; *A*(1), estimated density of test strain at time 1 d; *M*(1), estimated density of marker strain at time 1 d; ln, natural logarithm (logarithm to the base *e*).

### Mathematical Model

We used a simple transmission model to examine the qualitative effect of toxicity-dependent probabilities of *S*. *aureus* to cause invasive disease (see S4 Fig for a flow diagram). We considered two different strains, distinguished by their level of toxicity (high, *h*, and low, *l*), and assumed that susceptible individuals, *S*, become colonised with strain *i* (*I* = *h*,*l*) upon contact with either colonised or infected individuals (*C*_*i*_ and *I*_*i*_) with transmission rates *β*_*c*_ and *β*_*i*_, respectively. Individuals transition from colonisation to infection (i.e., SSTI) at a rate *δ*_*i*_, from which they either recover (at rate *τ*_*i*_ and *ρ*_*i*_, accounting for both treatment and immune mediated clearance) or go on to develop invasive diseases (bacteraemia, *B*_*i*_), with probability *σ*_*i*_, which we assume does not contribute to transmission. The model can then be given by the following set of differential equations for the proportions of the population being susceptible, colonised, infected, or suffering from invasive disease:

$$\frac{dS}{dt}=\mu -S(\lambda_{l}+\lambda_{h})+\tau_{l}I_{l}+\tau_{h}I_{h}-\mu S$$

$$\frac{dC_{h}}{dt}=\lambda_{h}S+\varrho_{h}I_{h}-(\mu +\delta_{h})C_{h}-\nu C_{h}$$

$$\frac{dC_{l}}{dt}=\lambda_{l}S+\varrho_{l}I_{l}-(\mu +\delta_{l})C_{l}+\nu C_{h}$$

$$\frac{dI_{h}}{dt}=\delta_{h}C_{h}-\varrho_{h}I_{h}-(\mu +\sigma_{h}+\tau_{h})I_{h}-\nu I_{h}$$

$$\frac{dI_{l}}{dt}=\delta_{l}C_{l}-\varrho_{l}I_{l}-(\mu +\sigma_{l}+\tau_{l})I_{l}+\nu I_{h}$$

$$\frac{dB_{i}}{dt}=\sigma_{i}I_{i}-(\mu +\chi )B_{i},\;i=h,l$$

with the force of infection of strain *i*, *λ*_*i*_ = *β*_*C*_ *C*_*i*_ *+ β*_*i*_ *I*_*i*._, disease-induced mortality *χ*, and *μ* as the natural birth/death rate. For simplicity, we did not allow for co- or superinfections but considered within-host evolution whereby more toxic strains can mutate (at a rate *ν*) towards lower levels of toxicity. Within this system, the probability of colonised individuals to develop infections (SSTI’s) was assumed to be positively correlated with the strain’s degree of toxicity (with *δ*_*h*_ > *δ*_*l*_), as were the transmission and treatment rates of infected individuals (i.e., *β*_*h*_ > *β*_*l*_, *τ*_*h*_ > *τ*_*l*_). For illustration purposes only, we assumed that when the strains have equal probabilities to develop invasive disease (i.e., *σ*_*1*_ = σ_*2*_), the less toxic strain has a higher fitness than the more toxic one. That is, we assumed that the less toxic strain is at an optimum, whereby toxicity-driven increases in transmissibility would be offset by higher clearance rates. Unless stated otherwise, we used the following parameter values: *μ* = 0.017, *β*_*C*_ = 0.05, *β*_*l*_ = 4, *β*_*h*_ = 4.4, *δ*_*l*_ = 2, *δ*_*h*_ = 2.2, *τ*_*l*_ = 3, *τ*_*h*_ = 3.3, *ρ*_*l*_ = *ρ*_*h*_ = 10, *ν* = 0.002, *χ* = 5.

## Supporting Information

S1 Data A file containing three spreadsheets with the toxicity data for the clinical isolates used in this study, illustrated in Fig 1. (XLSX)

S2 Data A spreadsheet containing the toxicity data for the transposon mutants used in this study, illustrated in Fig 2. (XLSX)

S3 Data An excel file containing five spreadsheets with the cell invasion, NETs, biofilm, hNP1 resistance, and protease activity for subset of isolates, illustrated in Fig 4. (XLSX)

S4 Data A spreadsheet containing the relative fitness data for the isolates tested here with and without human serum, illustrated in Fig 5. (XLSX)

S5 Data An excel file containing two spreadsheets with the toxicity data for a range of *S*. *aureus* toxin mutants and purified toxins; and comparing toxicity using T2 cells and fresh human neutrophils, illustrated in S1 Fig. (XLSX)

S6 Data A spreadsheet containing growth dynamic data for high- and low-toxicity isolates with and without human serum, illustrated in S2 Fig. (XLSX)

S1 Fig T2 cell susceptibility to *S*. *aureus* cytolytic toxins. A: Using a combination of isogenic mutant strains and purified toxins, the susceptibility of the T2 cell line is illustrated. Mean of six replicates are presented, error bars represent the 95% confidence intervals, * indicates statistically significant differences. B: The toxicity to both T2 cells and neutrophils of subset of six high- and six low-toxicity isolates from the single-patient collection was quantified. The effect of the low-toxicity isolates on cell death does not vary when fresh human neutrophils that are susceptible to LukAB and LukED are used. To access this data, see S5 Data.
(TIF)

S2 Fig Growth dynamics of 10 high- and 10 low-toxicity isolates in BHI with and without 5% (vol/vol) human serum. To access this data, see S6 Data.
(TIF)

S3 Fig Toxicity- and bacteraemia-dependent fitness landscape. A strain's fitness, here shown as its force of infection at equilibrium, is determined by its level of toxicity and its propensity to cause bacteraemia. Due to an evolutionary trade-off between toxicity-driven increase in transmissibility and treatment rate, fitness initially increases with enhanced toxicity but then declines as individuals become more likely to seek treatment faster, thus limiting the opportunity for onward transmission. With equal probabilities to cause bacteraemia (scenario 1), a more toxic strain (red) can therefore be outcompeted by a strain with lower levels of toxicity (green). In contrast, by assuming an inverse correlation between toxicity and the probability of causing bacteraemia (scenario 2), the more toxic strain can gain a competitive advantage, leading to the exclusion of the less toxic strain.
(TIF)

S4 Fig Flow diagram illustrating the dynamical processes underlying the mathematical model. The population is subdivided into different classes representing those susceptible (*S*), colonised with strain *i* (C_i), infected (SSTI) with strain i (*I_i*), and those bacteraemic (*B_i*).
(TIF)

S1 Table *S*. *aureus* isolates used in the study. (DOCX)

S2 Table List of SNPs associated with changes in toxicity as determined by PLINK. SNP site: number corresponds to the distance from the origin of replication. Gene: gene in which the toxicity-associated SNP resides. Description: known activity of the gene. AA change: amino acid change caused by the SNP. Tn mutant: name of the Tn mutant obtained from the Nebraska library in the relevant gene. Bold font indicates Tn mutants with functionally verified effect on toxicity.
(DOCX)

S3 Table Clinical features of the bacteraemic isolates used in this study. (DOCX)

## Abbreviations:

BHI: brain–heart infusion broth
ENC: early nose culture
GWAS: genome-wide association study
LBC: late blood culture
LNC: late nose culture
NET: neutrophil extracellular trap
PSM: phenol soluble modulins
PVL: Panton-Valentine-leukocidin
SSTI: skin and soft tissue infection
