# Supplementary figures and images for: Evolutionary Trade-Offs Underlie the Multi-faceted Virulence of Staphylococcus aureus
Source: PLoS Biol. 2015 Sep 2;13(9):e1002229. doi: 10.1371/journal.pbio.1002229 (PMC4558032; doi:10.1371/journal.pbio.1002229)

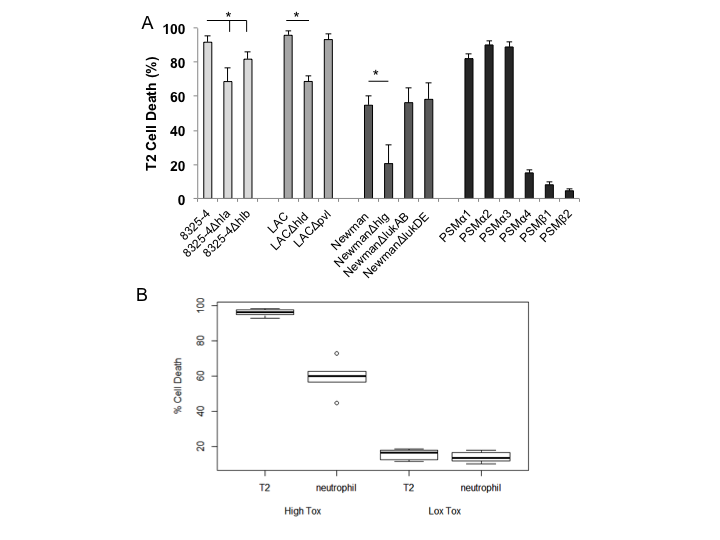

Supplement: S1 Fig — A: Using a combination of isogenic mutant strains and purified toxins, the susceptibility of the T2 cell line is illustrated. Mean of six replicates are presented, error bars represent the 95% confidence intervals, * indicates statistically significant differences. B: The toxicity to both T2 cells and neutrophils of subset of six high- and six low-toxicity isolates from the single-patient collection was quantified. The effect of the low-toxicity isolates on cell death does not vary when fresh human neutrophils that are susceptible to LukAB and LukED are used. To access this data, see S5 Data. (TIF) [file pbio.1002229.s007.tif]

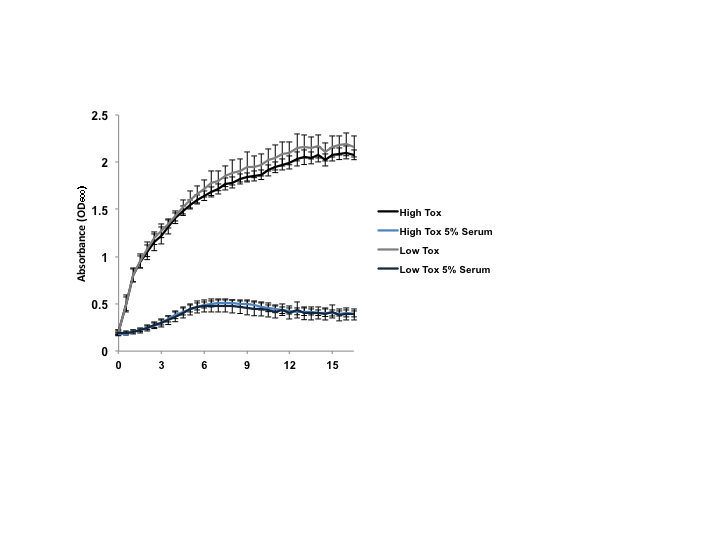

Supplement: S2 Fig — To access this data, see S6 Data. (TIF) [file pbio.1002229.s008.tif]

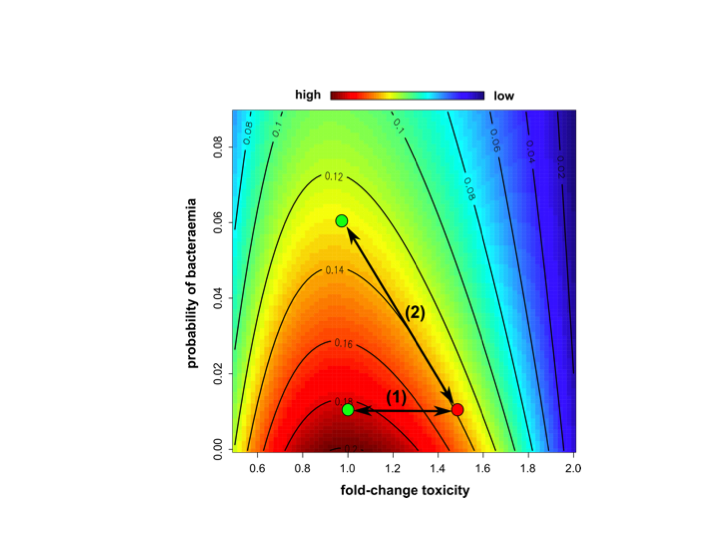

Supplement: S3 Fig — A strain's fitness, here shown as its force of infection at equilibrium, is determined by its level of toxicity and its propensity to cause bacteraemia. Due to an evolutionary trade-off between toxicity-driven increase in transmissibility and treatment rate, fitness initially increases with enhanced toxicity but then declines as individuals become more likely to seek treatment faster, thus limiting the opportunity for onward transmission. With equal probabilities to cause bacteraemia (scenario 1), a more toxic strain (red) can therefore be outcompeted by a strain with lower levels of toxicity (green). In contrast, by assuming an inverse correlation between toxicity and the probability of causing bacteraemia (scenario 2), the more toxic strain can gain a competitive advantage, leading to the exclusion of the less toxic strain. (TIF) [file pbio.1002229.s009.tif]

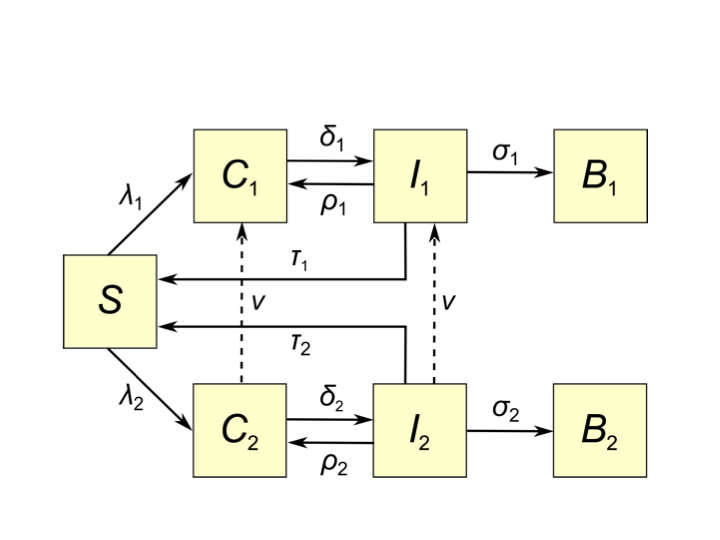

Supplement: S4 Fig — The population is subdivided into different classes representing those susceptible (S), colonised with strain i (C_i), infected (SSTI) with strain i (I_i), and those bacteraemic (B_i). (TIF) [file pbio.1002229.s010.tif]
