# Supplementary material for: Evolutionary Trade-Offs Underlie the Multi-faceted Virulence of Staphylococcus aureus
Source: PLoS Biol. 2015 Sep 2;13(9):e1002229. doi: 10.1371/journal.pbio.1002229 (PMC4558032; doi:10.1371/journal.pbio.1002229)
Supplement: S1 Table — (DOCX) [file pbio.1002229.s011.docx]

| **Isolate** | **Genotype** | **Infectious source** | **Reference or ENA accession no.** |
| --- | --- | --- | --- |
| MR005 | Wildtype clinical USA300 | Blood (bacteraemia) | ERR449858 |
| MR007 | Wildtype clinical USA300 | Blood (bacteraemia) | ERR449859 |
| MR018 | Wildtype clinical USA300 | Blood (bacteraemia) | ERR449860 |
| MR019 | Wildtype clinical USA300 | Blood (bacteraemia) | ERR449861 |
| MR021 | Wildtype clinical USA300 | Blood (bacteraemia) | ERR449862 |
| MR022 | Wildtype clinical USA300 | Blood (bacteraemia) | ERR449863 |
| MR023 | Wildtype clinical USA300 | Blood (bacteraemia) | ERR449864 |
| MR025 | Wildtype clinical USA300 | Blood (bacteraemia) | ERR449865 |
| MR026 | Wildtype clinical USA300 | Blood (bacteraemia) | ERR449866 |
| MR027 | Wildtype clinical USA300 | Blood (bacteraemia) | ERR449867 |
| MR029 | Wildtype clinical USA300 | Blood (bacteraemia) | ERR449868 |
| MR030 | Wildtype clinical USA300 | Blood (bacteraemia) | ERR449869 |
| MR031 | Wildtype clinical USA300 | Blood (bacteraemia) | ERR449870 |
| MR035 | Wildtype clinical USA300 | Blood (bacteraemia) | ERR449871 |
| MR036 | Wildtype clinical USA300 | Blood (bacteraemia) | ERR449872 |
| MR039 | Wildtype clinical USA300 | Blood (bacteraemia) | ERR449873 |
| MR047 | Wildtype clinical USA300 | Blood (bacteraemia) | ERR449874 |
| MR051 | Wildtype clinical USA300 | Blood (bacteraemia) | ERR449875 |
| MR060 | Wildtype clinical USA300 | Blood (bacteraemia) | ERR449876 |
| MR063 | Wildtype clinical USA300 | Blood (bacteraemia) | ERR449877 |
| MR064 | Wildtype clinical USA300 | Blood (bacteraemia) | ERR449878 |
| MR065 | Wildtype clinical USA300 | Blood (bacteraemia) | ERR449879 |
| MR072 | Wildtype clinical USA300 | Blood (bacteraemia) | ERR449880 |
| MR073 | Wildtype clinical USA300 | Blood (bacteraemia) | ERR449881 |
| MR074 | Wildtype clinical USA300 | Blood (bacteraemia) | ERR449882 |
| MR077 | Wildtype clinical USA300 | Blood (bacteraemia) | ERR449883 |
| MR078 | Wildtype clinical USA300 | Blood (bacteraemia) | ERR449884 |
| MR081 | Wildtype clinical USA300 | Blood (bacteraemia) | ERR449885 |
| MR083 | Wildtype clinical USA300 | Blood (bacteraemia) | ERR449886 |
| MR084 | Wildtype clinical USA300 | Blood (bacteraemia) | ERR449887 |
| MR087 | Wildtype clinical USA300 | Blood (bacteraemia) | ERR449888 |
| MR090 | Wildtype clinical USA300 | Blood (bacteraemia) | ERR449889 |
| MR091 | Wildtype clinical USA300 | Blood (bacteraemia) | ERR449890 |
| MR096 | Wildtype clinical USA300 | Blood (bacteraemia) | ERR449891 |
| MR107 | Wildtype clinical USA300 | Blood (bacteraemia) | ERR449892 |
| MR110 | Wildtype clinical USA300 | Blood (bacteraemia) | ERR449893 |
| USFL008 | Wildtype clinical USA300 | Nose/skin (carriage) | [^1^](#_ENREF_1) |
| USFL009 | Wildtype clinical USA300 | Nose/skin (carriage) | [^1^](#_ENREF_1) |
| USFL012 | Wildtype clinical USA300 | Nose/skin (carriage) | [^1^](#_ENREF_1) |
| USFL028 | Wildtype clinical USA300 | Nose/skin (carriage) | [^1^](#_ENREF_1) |
| USFL042 | Wildtype clinical USA300 | Nose/skin (carriage) | [^1^](#_ENREF_1) |
| USFL061 | Wildtype clinical USA300 | Nose/skin (carriage) | [^1^](#_ENREF_1) |
| USFL063 | Wildtype clinical USA300 | Nose/skin (carriage) | [^1^](#_ENREF_1) |
| USFL074 | Wildtype clinical USA300 | Nose/skin (carriage) | [^1^](#_ENREF_1) |
| USFL077 | Wildtype clinical USA300 | Nose/skin (carriage) | [^1^](#_ENREF_1) |
| USFL082 | Wildtype clinical USA300 | Nose/skin (carriage) | [^1^](#_ENREF_1) |
| USFL093 | Wildtype clinical USA300 | Nose/skin (carriage) | [^1^](#_ENREF_1) |
| USFL119 | Wildtype clinical USA300 | Nose/skin (carriage) | [^1^](#_ENREF_1) |
| USFL130 | Wildtype clinical USA300 | Nose/skin (carriage) | [^1^](#_ENREF_1) |
| USFL141 | Wildtype clinical USA300 | Nose/skin (carriage) | [^1^](#_ENREF_1) |
| USFL153 | Wildtype clinical USA300 | Nose/skin (carriage) | [^1^](#_ENREF_1) |
| USFL156 | Wildtype clinical USA300 | Nose/skin (carriage) | [^1^](#_ENREF_1) |
| USFL166 | Wildtype clinical USA300 | Nose/skin (carriage) | [^1^](#_ENREF_1) |
| USFL167 | Wildtype clinical USA300 | Nose/skin (carriage) | [^1^](#_ENREF_1) |
| USFL169 | Wildtype clinical USA300 | Nose/skin (carriage) | [^1^](#_ENREF_1) |
| USFL182 | Wildtype clinical USA300 | Nose/skin (carriage) | [^1^](#_ENREF_1) |
| USFL200 | Wildtype clinical USA300 | Nose/skin (carriage) | [^1^](#_ENREF_1) |
| USFL211 | Wildtype clinical USA300 | Nose/skin (carriage) | [^1^](#_ENREF_1) |
| USFL213 | Wildtype clinical USA300 | Nose/skin (carriage) | [^1^](#_ENREF_1) |
| USFL224 | Wildtype clinical USA300 | Nose/skin (carriage) | [^1^](#_ENREF_1) |
| USFL225 | Wildtype clinical USA300 | Nose/skin (carriage) | [^1^](#_ENREF_1) |
| USFL230 | Wildtype clinical USA300 | Nose/skin (carriage) | [^1^](#_ENREF_1) |
| USFL231 | Wildtype clinical USA300 | Nose/skin (carriage) | [^1^](#_ENREF_1) |
| USFL243 | Wildtype clinical USA300 | Nose/skin (carriage) | [^1^](#_ENREF_1) |
| USFL248 | Wildtype clinical USA300 | Nose/skin (carriage) | [^1^](#_ENREF_1) |
| USFL259 | Wildtype clinical USA300 | Nose/skin (carriage) | [^1^](#_ENREF_1) |
| USFL263 | Wildtype clinical USA300 | Nose/skin (carriage) | [^1^](#_ENREF_1) |
| USFL267 | Wildtype clinical USA300 | Nose/skin (carriage) | [^1^](#_ENREF_1) |
| USFL269 | Wildtype clinical USA300 | Nose/skin (carriage) | [^1^](#_ENREF_1) |
| USFL271 | Wildtype clinical USA300 | Nose/skin (carriage) | [^1^](#_ENREF_1) |
| USFL272 | Wildtype clinical USA300 | Nose/skin (carriage) | [^1^](#_ENREF_1) |
| USFL302 | Wildtype clinical USA300 | Nose/skin (carriage) | [^1^](#_ENREF_1) |
| USFL303 | Wildtype clinical USA300 | Nose/skin (carriage) | [^1^](#_ENREF_1) |
| USFL304 | Wildtype clinical USA300 | Nose/skin (carriage) | [^1^](#_ENREF_1) |
| USFL016 | Wildtype clinical USA300 | Abscess (SSTI) | [^1^](#_ENREF_1) |
| USFL018 | Wildtype clinical USA300 | Abscess (SSTI) | [^1^](#_ENREF_1) |
| USFL020 | Wildtype clinical USA300 | Abscess (SSTI) | [^1^](#_ENREF_1) |
| USFL021 | Wildtype clinical USA300 | Abscess (SSTI) | [^1^](#_ENREF_1) |
| USFL034 | Wildtype clinical USA300 | Abscess (SSTI) | [^1^](#_ENREF_1) |
| USFL035 | Wildtype clinical USA300 | Abscess (SSTI) | [^1^](#_ENREF_1) |
| USFL036 | Wildtype clinical USA300 | Abscess (SSTI) | [^1^](#_ENREF_1) |
| USFL039 | Wildtype clinical USA300 | Abscess (SSTI) | [^1^](#_ENREF_1) |
| USFL056 | Wildtype clinical USA300 | Abscess (SSTI) | [^1^](#_ENREF_1) |
| USFL057 | Wildtype clinical USA300 | Abscess (SSTI) | [^1^](#_ENREF_1) |
| USFL059 | Wildtype clinical USA300 | Abscess (SSTI) | [^1^](#_ENREF_1) |
| USFL069 | Wildtype clinical USA300 | Abscess (SSTI) | [^1^](#_ENREF_1) |
| USFL095 | Wildtype clinical USA300 | Abscess (SSTI) | [^1^](#_ENREF_1) |
| USFL097 | Wildtype clinical USA300 | Abscess (SSTI) | [^1^](#_ENREF_1) |
| USFL103 | Wildtype clinical USA300 | Abscess (SSTI) | [^1^](#_ENREF_1) |
| USFL110 | Wildtype clinical USA300 | Abscess (SSTI) | [^1^](#_ENREF_1) |
| USFL111 | Wildtype clinical USA300 | Abscess (SSTI) | [^1^](#_ENREF_1) |
| USFL113 | Wildtype clinical USA300 | Abscess (SSTI) | [^1^](#_ENREF_1) |
| USFL136 | Wildtype clinical USA300 | Abscess (SSTI) | [^1^](#_ENREF_1) |
| USFL137 | Wildtype clinical USA300 | Abscess (SSTI) | [^1^](#_ENREF_1) |
| USFL138 | Wildtype clinical USA300 | Abscess (SSTI) | [^1^](#_ENREF_1) |
| USFL139 | Wildtype clinical USA300 | Abscess (SSTI) | [^1^](#_ENREF_1) |
| USFL149 | Wildtype clinical USA300 | Abscess (SSTI) | [^1^](#_ENREF_1) |
| USFL152 | Wildtype clinical USA300 | Abscess (SSTI) | [^1^](#_ENREF_1) |
| USFL158 | Wildtype clinical USA300 | Abscess (SSTI) | [^1^](#_ENREF_1) |
| USFL159 | Wildtype clinical USA300 | Abscess (SSTI) | [^1^](#_ENREF_1) |
| USFL160 | Wildtype clinical USA300 | Abscess (SSTI) | [^1^](#_ENREF_1) |
| USFL162 | Wildtype clinical USA300 | Abscess (SSTI) | [^1^](#_ENREF_1) |
| USFL164 | Wildtype clinical USA300 | Abscess (SSTI) | [^1^](#_ENREF_1) |
| USFL165 | Wildtype clinical USA300 | Abscess (SSTI) | [^1^](#_ENREF_1) |
| USFL173 | Wildtype clinical USA300 | Abscess (SSTI) | [^1^](#_ENREF_1) |
| USFL174 | Wildtype clinical USA300 | Abscess (SSTI) | [^1^](#_ENREF_1) |
| USFL194 | Wildtype clinical USA300 | Abscess (SSTI) | [^1^](#_ENREF_1) |
| USFL198 | Wildtype clinical USA300 | Abscess (SSTI) | [^1^](#_ENREF_1) |
| USFL218 | Wildtype clinical USA300 | Abscess (SSTI) | [^1^](#_ENREF_1) |
| USFL219 | Wildtype clinical USA300 | Abscess (SSTI) | [^1^](#_ENREF_1) |
| USFL220 | Wildtype clinical USA300 | Abscess (SSTI) | [^1^](#_ENREF_1) |
| USFL221 | Wildtype clinical USA300 | Abscess (SSTI) | [^1^](#_ENREF_1) |
| USFL222 | Wildtype clinical USA300 | Abscess (SSTI) | [^1^](#_ENREF_1) |
| USFL223 | Wildtype clinical USA300 | Abscess (SSTI) | [^1^](#_ENREF_1) |
| USFL237 | Wildtype clinical USA300 | Abscess (SSTI) | [^1^](#_ENREF_1) |
| USFL239 | Wildtype clinical USA300 | Abscess (SSTI) | [^1^](#_ENREF_1) |
| USFL240 | Wildtype clinical USA300 | Abscess (SSTI) | [^1^](#_ENREF_1) |
| USFL250 | Wildtype clinical USA300 | Abscess (SSTI) | [^1^](#_ENREF_1) |
| USFL255 | Wildtype clinical USA300 | Abscess (SSTI) | [^1^](#_ENREF_1) |
| USFL256 | Wildtype clinical USA300 | Abscess (SSTI) | [^1^](#_ENREF_1) |
| USFL258 | Wildtype clinical USA300 | Abscess (SSTI) | [^1^](#_ENREF_1) |
| USFL273 | Wildtype clinical USA300 | Abscess (SSTI) | [^1^](#_ENREF_1) |
| USFL274 | Wildtype clinical USA300 | Abscess (SSTI) | [^1^](#_ENREF_1) |
| USFL276 | Wildtype clinical USA300 | Abscess (SSTI) | [^1^](#_ENREF_1) |
| USFL277 | Wildtype clinical USA300 | Abscess (SSTI) | [^1^](#_ENREF_1) |
| USFL279 | Wildtype clinical USA300 | Abscess (SSTI) | [^1^](#_ENREF_1) |
| USFL282 | Wildtype clinical USA300 | Abscess (SSTI) | [^1^](#_ENREF_1) |
| USFL319 | Wildtype clinical USA300 | Abscess (SSTI) | [^1^](#_ENREF_1) |
| USFL320 | Wildtype clinical USA300 | Abscess (SSTI) | [^1^](#_ENREF_1) |
| USFL326 | Wildtype clinical USA300 | Abscess (SSTI) | [^1^](#_ENREF_1) |
| USFL327 | Wildtype clinical USA300 | Abscess (SSTI) | [^1^](#_ENREF_1) |
| USFL330 | Wildtype clinical USA300 | Abscess (SSTI) | [^1^](#_ENREF_1) |
| USFL339 | Wildtype clinical USA300 | Abscess (SSTI) | [^1^](#_ENREF_1) |
| USFL341 | Wildtype clinical USA300 | Abscess (SSTI) | [^1^](#_ENREF_1) |
| ENC1 384-16 | Wildtype clinical ST15 | Nose (carriage) | [^2^](#_ENREF_2) |
| ENC1 384-21 | Wildtype clinical ST15 | Nose (carriage) | [^2^](#_ENREF_2) |
| ENC1 384-22 | Wildtype clinical ST15 | Nose (carriage) | [^2^](#_ENREF_2) |
| ENC1 384-45 | Wildtype clinical ST15 | Nose (carriage) | [^2^](#_ENREF_2) |
| ENC1 384-56 | Wildtype clinical ST15 | Nose (carriage) | [^2^](#_ENREF_2) |
| ENC1 384-57 | Wildtype clinical ST15 | Nose (carriage) | [^2^](#_ENREF_2) |
| ENC1 384-59 | Wildtype clinical ST15 | Nose (carriage) | [^2^](#_ENREF_2) |
| ENC1 384-62 | Wildtype clinical ST15 | Nose (carriage) | [^2^](#_ENREF_2) |
| ENC1 384-79 | Wildtype clinical ST15 | Nose (carriage) | [^2^](#_ENREF_2) |
| ENC1 384-81 | Wildtype clinical ST15 | Nose (carriage) | [^2^](#_ENREF_2) |
| ENC1 384-86 | Wildtype clinical ST15 | Nose (carriage) | [^2^](#_ENREF_2) |
| ENC1 384-89 | Wildtype clinical ST15 | Nose (carriage) | [^2^](#_ENREF_2) |
| ENC2 384-27 | Wildtype clinical ST15 | Nose (carriage) | [^2^](#_ENREF_2) |
| ENC2 384-32 | Wildtype clinical ST15 | Nose (carriage) | [^2^](#_ENREF_2) |
| ENC2 384-35 | Wildtype clinical ST15 | Nose (carriage) | [^2^](#_ENREF_2) |
| ENC2 384-36 | Wildtype clinical ST15 | Nose (carriage) | [^2^](#_ENREF_2) |
| ENC2 384-50 | Wildtype clinical ST15 | Nose (carriage) | [^2^](#_ENREF_2) |
| ENC2 384-54 | Wildtype clinical ST15 | Nose (carriage) | [^2^](#_ENREF_2) |
| ENC2 384-63 | Wildtype clinical ST15 | Nose (carriage) | [^2^](#_ENREF_2) |
| ENC2 384-67 | Wildtype clinical ST15 | Nose (carriage) | [^2^](#_ENREF_2) |
| ENC2 384-68 | Wildtype clinical ST15 | Nose (carriage) | [^2^](#_ENREF_2) |
| ENC2 384-80 | Wildtype clinical ST15 | Nose (carriage) | [^2^](#_ENREF_2) |
| ENC2 384-84 | Wildtype clinical ST15 | Nose (carriage) | [^2^](#_ENREF_2) |
| ENC2 384-88 | Wildtype clinical ST15 | Nose (carriage) | [^2^](#_ENREF_2) |
| ENC4 384-28 | Wildtype clinical ST15 | Nose (carriage) | [^2^](#_ENREF_2) |
| ENC4 384-29 | Wildtype clinical ST15 | Nose (carriage) | [^2^](#_ENREF_2) |
| ENC4 384-38 | Wildtype clinical ST15 | Nose (carriage) | [^2^](#_ENREF_2) |
| ENC4 384-48 | Wildtype clinical ST15 | Nose (carriage) | [^2^](#_ENREF_2) |
| ENC4 384-49 | Wildtype clinical ST15 | Nose (carriage) | [^2^](#_ENREF_2) |
| ENC4 384-51 | Wildtype clinical ST15 | Nose (carriage) | [^2^](#_ENREF_2) |
| ENC4 384-60 | Wildtype clinical ST15 | Nose (carriage) | [^2^](#_ENREF_2) |
| ENC4 384-61 | Wildtype clinical ST15 | Nose (carriage) | [^2^](#_ENREF_2) |
| ENC4 384-66 | Wildtype clinical ST15 | Nose (carriage) | [^2^](#_ENREF_2) |
| ENC4 384-70 | Wildtype clinical ST15 | Nose (carriage) | [^2^](#_ENREF_2) |
| ENC4 384-72 | Wildtype clinical ST15 | Nose (carriage) | [^2^](#_ENREF_2) |
| ENC4 384-73 | Wildtype clinical ST15 | Nose (carriage) | [^2^](#_ENREF_2) |
| ENC6 384-13 | Wildtype clinical ST15 | Nose (carriage) | [^2^](#_ENREF_2) |
| ENC6 384-15 | Wildtype clinical ST15 | Nose (carriage) | [^2^](#_ENREF_2) |
| ENC6 384-19 | Wildtype clinical ST15 | Nose (carriage) | [^2^](#_ENREF_2) |
| ENC6 384-24 | Wildtype clinical ST15 | Nose (carriage) | [^2^](#_ENREF_2) |
| ENC6 384-26 | Wildtype clinical ST15 | Nose (carriage) | [^2^](#_ENREF_2) |
| ENC6 384-39 | Wildtype clinical ST15 | Nose (carriage) | [^2^](#_ENREF_2) |
| ENC6 384-41 | Wildtype clinical ST15 | Nose (carriage) | [^2^](#_ENREF_2) |
| ENC6 384-44 | Wildtype clinical ST15 | Nose (carriage) | [^2^](#_ENREF_2) |
| ENC6 384-46 | Wildtype clinical ST15 | Nose (carriage) | [^2^](#_ENREF_2) |
| ENC6 384-55 | Wildtype clinical ST15 | Nose (carriage) | [^2^](#_ENREF_2) |
| ENC6 384-69 | Wildtype clinical ST15 | Nose (carriage) | [^2^](#_ENREF_2) |
| ENC6 384-74 | Wildtype clinical ST15 | Nose (carriage) | [^2^](#_ENREF_2) |
| ENC8 384-01 | Wildtype clinical ST15 | Nose (carriage) | [^2^](#_ENREF_2) |
| ENC8 384-04 | Wildtype clinical ST15 | Nose (carriage) | [^2^](#_ENREF_2) |
| ENC8 384-05 | Wildtype clinical ST15 | Nose (carriage) | [^2^](#_ENREF_2) |
| ENC8 384-10 | Wildtype clinical ST15 | Nose (carriage) | [^2^](#_ENREF_2) |
| ENC8 384-14 | Wildtype clinical ST15 | Nose (carriage) | [^2^](#_ENREF_2) |
| ENC8 384-17 | Wildtype clinical ST15 | Nose (carriage) | [^2^](#_ENREF_2) |
| ENC8 384-20 | Wildtype clinical ST15 | Nose (carriage) | [^2^](#_ENREF_2) |
| ENC8 384-33 | Wildtype clinical ST15 | Nose (carriage) | [^2^](#_ENREF_2) |
| ENC8 384-53 | Wildtype clinical ST15 | Nose (carriage) | [^2^](#_ENREF_2) |
| ENC8 384-65 | Wildtype clinical ST15 | Nose (carriage) | [^2^](#_ENREF_2) |
| ENC8 384-82 | Wildtype clinical ST15 | Nose (carriage) | [^2^](#_ENREF_2) |
| ENC8 384-83 | Wildtype clinical ST15 | Nose (carriage) | [^2^](#_ENREF_2) |
| LNC12 384-02 | Wildtype clinical ST15 | Nose (carriage) | [^2^](#_ENREF_2) |
| LNC12 384-03 | Wildtype clinical ST15 | Nose (carriage) | [^2^](#_ENREF_2) |
| LNC12 384-08 | Wildtype clinical ST15 | Nose (carriage) | [^2^](#_ENREF_2) |
| LNC12 384-09 | Wildtype clinical ST15 | Nose (carriage) | [^2^](#_ENREF_2) |
| LNC12 384-11 | Wildtype clinical ST15 | Nose (carriage) | [^2^](#_ENREF_2) |
| LNC12 384-23 | Wildtype clinical ST15 | Nose (carriage) | [^2^](#_ENREF_2) |
| LNC12 384-30 | Wildtype clinical ST15 | Nose (carriage) | [^2^](#_ENREF_2) |
| LNC12 384-47 | Wildtype clinical ST15 | Nose (carriage) | [^2^](#_ENREF_2) |
| LNC12 384-75 | Wildtype clinical ST15 | Nose (carriage) | [^2^](#_ENREF_2) |
| LNC12 384-76 | Wildtype clinical ST15 | Nose (carriage) | [^2^](#_ENREF_2) |
| LNC12 384-77 | Wildtype clinical ST15 | Nose (carriage) | [^2^](#_ENREF_2) |
| LNC12 384-87 | Wildtype clinical ST15 | Nose (carriage) | [^2^](#_ENREF_2) |
| LBC15 384-12 | Wildtype clinical ST15 | Blood (bacteraemia) | [^2^](#_ENREF_2) |
| LBC15 384-18 | Wildtype clinical ST15 | Blood (bacteraemia) | [^2^](#_ENREF_2) |
| LBC15 384-25 | Wildtype clinical ST15 | Blood (bacteraemia) | [^2^](#_ENREF_2) |
| LBC15 384-31 | Wildtype clinical ST15 | Blood (bacteraemia) | [^2^](#_ENREF_2) |
| LBC15 384-37 | Wildtype clinical ST15 | Blood (bacteraemia) | [^2^](#_ENREF_2) |
| LBC15 384-42 | Wildtype clinical ST15 | Blood (bacteraemia) | [^2^](#_ENREF_2) |
| LBC15 384-43 | Wildtype clinical ST15 | Blood (bacteraemia) | [^2^](#_ENREF_2) |
| LBC15 384-52 | Wildtype clinical ST15 | Blood (bacteraemia) | [^2^](#_ENREF_2) |
| LBC15 384-64 | Wildtype clinical ST15 | Blood (bacteraemia) | [^2^](#_ENREF_2) |
| LBC15 384-71 | Wildtype clinical ST15 | Blood (bacteraemia) | [^2^](#_ENREF_2) |
| LBC15 384-78 | Wildtype clinical ST15 | Blood (bacteraemia) | [^2^](#_ENREF_2) |
| JE2 | USA300 isolate laboratory strain | N/A | [^3^](#_ENREF_3) |
| NE95 | JE2 Tn::*agrB* | N/A | [^3^](#_ENREF_3) |
| NE348 | JE2 Tn::*ftsK* | N/A | [^3^](#_ENREF_3) |
| NE1647 | JE2 Tn::*rpsA* | N/A | [^3^](#_ENREF_3) |
| NE1686 | JE2 Tn::SAUSA300_0750 | N/A | [^3^](#_ENREF_3) |
| NE1770 | JE2 Tn::*sucD* | N/A | [^3^](#_ENREF_3) |
| NE699 | JE2 Tn::*clpC* | N/A | [^3^](#_ENREF_3) |
| NE1304 | JE2 Tn::SAUSA300_2326 | N/A | [^3^](#_ENREF_3) |
| MSSA466 | Wild type clinical ST8 Tet^r^ | Abscess (SSTI) | ^4^ |

1. Uhlemann, A.C. *et al.* Molecular tracing of the emergence, diversification, and transmission of S. aureus sequence type 8 in a New York community. *Proc Natl Acad Sci U S A* **111**, 6738-43 (2014).

2. Young, B.C. *et al.* Evolutionary dynamics of Staphylococcus aureus during progression from carriage to disease. *Proc Natl Acad Sci U S A* **109**, 4550-5 (2012).

3. Fey, P.D. *et al.* A genetic resource for rapid and comprehensive phenotype screening of nonessential Staphylococcus aureus genes. *MBio* **4**, e00537-12 (2013).

4. Peacock et al. Virulent combinations of adhesin and toxin genes in natural populations of Staphylococcus aureus. *Infect Immun.* 70:4987-96 (2002).

.
