# Supplementary material for: Evolutionary Trade-Offs Underlie the Multi-faceted Virulence of Staphylococcus aureus
Source: PLoS Biol. 2015 Sep 2;13(9):e1002229. doi: 10.1371/journal.pbio.1002229 (PMC4558032; doi:10.1371/journal.pbio.1002229)
Supplement: S2 Table — SNP site: number corresponds to the distance from the origin of replication. Gene: gene in which the toxicity-associated SNP resides. Description: known activity of the gene. AA change: amino acid change caused by the SNP. Tn mutant: name of the Tn mutant obtained from the Nebraska library in the relevant gene. Bold font indicates Tn mutants with functionally verified effect on toxicity. (DOCX) [file pbio.1002229.s012.docx]

| **SNP site** | **Gene** | **Description** | **AA change** | **Tn mutant** |
| --- | --- | --- | --- | --- |
| 48890 | ccrA | cassette chromosome recombinase A | Met -> Thr | NE1396 |
| 121825 | SAUSA300_0108 | antigen, 67 kDa | Gly -> Asp | NE757 |
| 155472 | SAUSA300_0136 | cell wall surface anchor family protein | Thr -> Pro | NE1032 |
| 225815 | SAUSA300_0193 | conserved hypothetical protein | Thr -> Lys | NE1253 |
| 275048 | fadE | acyl-CoA synthetase FadE | Ala -> Thr | NE539 |
| 311680 | bglA | 6-phospho-beta-glucosidase | Gly -> Val | NE1584 |
| 335489 | SAUSA300_0282 | conserved hypothetical protein | Ile -> Thr | NE878 |
| 363677 | SAUSA300_0312 | indigoidine synthase family protein | Ala -> Val | NE1039 |
| 365895 | SAUSA300_0314 | sodium:solute symporter family protein | Ile -> Val | NE969 |
| 384695 | SAUSA300_0332 | PTS system, IIA component | Thr -> Ala | NE1282 |
| 396294 | SAUSA300_0344 | putative lipoprotein | Arg -> Cys | NE1559 |
| 402767 | SAUSA300_0352 | ABC transporter, ATP-binding protein | Pro -> Leu | NE756 |
| 409423 | metE | 5-methyltetrahydropteroyltriglutamate-- homocysteine S-methyltransferase | Trp -> Arg | NE944 |
| 498054 | gltB | glutamate synthase, large subunit | Leu -> Ser | NE390 |
| 529238 | SAUSA300_0469 | primase-related protein | Ala -> Val | NE905 |
| 549475 | SAUSA300_0489 | putative cell division protein FtsH | Ser -> Tyr | NE1313 |
| **571054** | **clpC** | **endopeptidase** | **Thr -> Ile** | **NE699** |
| 572497 | radA | DNA repair protein RadA | Pro -> Ser | NE1176 |
| 624215 | SAUSA300_0550 | glycosyl transferase, group 1 family protein | Ala -> Thr | NE105 |
| 627694 | SAUSA300_0553 | conserved hypothetical protein | Thr -> Ile | NE1165 |
| 664136 | SAUSA300_0590 | conserved hypothetical protein | Pro -> Gln | NE1402 |
| 688965 | SAUSA300_0617 | Na+/H+ antiporter | Phe -> Leu | NE1504 |
| 727942 | SAUSA300_0652 | putative membrane protein | Ala -> Val | NE1797 |
| 729481 | SAUSA300_0653 | transcriptional regulator, AraC family | Ala -> Val | NE872 |
| 740846 | SAUSA300_0667 | YaiI/YqxD family protein | Ser -> Thr | NE984 |
| 832870 | SAUSA300_0746 | TPR domain protein | Val -> Phe | NE490 |
| **837108** | **SAUSA300_0750** | **conserved hypothetical protein** | **Glu -> Asp** | **NE1686** |
| 847866 | gpmI | 2,3-bisphosphoglycerate-independent phosphoglycerate mutase | Tyr -> Phe | NE1891 |
| 871593 | SAUSA300_0784 | LysE/YggA family protein | Thr -> Ile | NE339 |
| 1001261 | SAUSA300_0913 | putative membrane protein | Asp -> Asn | NE1270 |
| 1064986 | purF | amidophosphoribosyltransferase | Gln -> Lys | NE581 |
| 1066640 | purM | phosphoribosylformylglycinamidine cyclo-ligase | Ala -> Thr | NE1101 |
| 1074815 | SAUSA300_0980 | putative membrane protein | Asp -> Asn | NE1044 |
| 1096101 | potB | spermidine/putrescine ABC transporter, permease protein | Ile -> Ser | NE1817 |
| 1149711 | SAUSA300_1051 | conserved hypothetical protein | Gly -> Asp | NE1711 |
| 1190133 | SAUSA300_1088 | glyoxalase family protein | Ser -> Asn | NE1832 |
| 1202002 | carB | carbamoyl-phosphate synthase, large subunit | Ser -> Tyr | NE1301 |
| **1248647** | **sucD** | **succinyl-CoA synthetase, alpha subunit** | **Ile -> Val** | **NE1770** |
| 1249125 | lytN | cell wall hydrolase | Phe -> Ile | NE402 |
| 1260381 | codY | GTP-sensing transcriptional pleiotropic repressor CodY | Val -> Ala | NE1555 |
| **1287966** | **ftsK** | **DNA translocase FtsK** | **His -> Asn** | **NE348** |
| 1320934 | SAUSA300_1199 | putative aluminium resistance protein | Ala -> Thr | NE1294 |
| 1346465 | thrB | homoserine kinase | Thr -> Ser | NE672 |
| 1422673 | SAUSA300_1291 | hippurate hydrolase | Phe -> Ser | NE1668 |
| 1426386 | SAUSA300_1296 | conserved hypothetical protein | Ala -> Pro | NE1793 |
| 1445934 | SAUSA300_1312 | acetyltransferase, GNAT family | Glu -> Lys | NE1634 |
| 1450394 | SAUSA300_1318 | DegV family protein | Ala -> Thr | NE403 |
| 1453673 | SAUSA300_1322 | conserved hypothetical protein | Pro -> Ala | NE1119 |
| 1462399 | SAUSA300_1327 | cell surface protein | Gln -> STOP | NE1 |
| **1532903** | **rpsA** | **30S ribosomal protein S1** | **Thr -> Ile** | **NE1647** |
| 1544401 | SAUSA300_1378 | conserved hypothetical protein | Ala -> Ser | NE1284 |
| 1549685 | SAUSA300_1383 | phiSLT ORF484-like protein, lysin | Val -> Ile | NE1655 |
| 1562314 | SAUSA300_1393 | phiSLT ORF2067-like protein, phage tail tape measure protein | Val ->Ala | NE47 |
| 1567798 | SAUSA300_1401 | phiSLT ORF387-like protein, putative phage capsid protein | Lys -> Stop | NE898 |
| 1569831 | SAUSA300_1403 | phiSLT ORF412-like protein, portal protein | Asn -> Ile | NE940 |
| 1574244 | SAUSA300_1408 | phage helicase | His -> Tyr | NE860 |
| 1576872 | SAUSA300_1410 | virulence-associated protein E | Glu -> Asp | NE783 |
| 1586212 | SAUSA300_1428 | conserved hypothetical phage protein | Asn -> Thr | NE1818 |
| 1652663 | gcvT | aminomethyltransferase (glycine cleavage system T protein) | Val -> Phe | NE660 |
| 1678619 | era | GTP-binding protein Era | Arg -> His | NE778 |
| 1693739 | SAUSA300_1543 | oxygen-independent coproporphyrinogen III oxidase | Thr -> Ala | NE555 |
| 1711026 | SAUSA300_1561 | putative membrane protein | Ala -> Val | NE1130 |
| 1740316 | lytH | N-acetylmuramoyl-L-alanine amidase | Gly -> Val | NE1369 |
| 1798320 | icd | isocitrate dehydrogenase, NADP-dependent | Ala -> Ser | NE491 |
| 1815532 | SAUSA300_1650 | conserved hypothetical protein | Pro -> Thr | NE1208 |
| 1834209 | SAUSA300_1669 | aminotransferase, class V | Ala -> Val | NE1652 |
| 1844577 | SAUSA300_1677 | cell wall surface anchor family protein | Thr -> Ile | NE1075 |
| 1847236 | fhs | formate-tetrahydrofolate ligase | Asp -> Tyr | NE706 |
| 1851284 | acuA | acetoin utilization protein AcuA | Phe -> Leu | NE1696 |
| 1889666 | SAUSA300_1706 | conserved hypothetical protein | Lys -> Glu | NE855 |
| 1908169 | SAUSA300_1725 | transaldolase | Ile -> Val | NE1170 |
| 1930744 | SAUSA300_1750 | conserved hypothetical protein | Asp -> Asn | NE703 |
| 1965351 | hemE | uroporphyrinogen decarboxylase | Thr -> Lys | NE1588 |
| 1968250 | SAUSA300_1785 | putative ABC transporter protein EcsB | Thr -> Ile | NE107 |
| 2004353 | SAUSA300_1843 | D-isomer specific 2-hydroxyacid dehydrogenase family protein | Thr -> Met | NE1460 |
| 2026967 | vraS | two-component sensor histidine kinase | Asp -> Tyr | NE823 |
| 2044766 | putP | high affinity proline permease | Gly -> Arg | NE889 |
| 2133403 | SAUSA300_1977 | conserved hypothetical protein | Glu -> Lys | NE502 |
| 2156607 | SAUSA300_1998 | putative membrane protein | Trp -> Arg | NE686 |
| 2170743 | leuA | 2-isopropylmalate synthase | Val -> Ile | NE1103 |
| 2173497 | leuC | 3-isopropylmalate dehydratase, large subunit | Ala -> Thr | NE875 |
| 2349683 | SAUSA300_2168 | conserved hypothetical protein | Ser -> Asn | NE1220 |
| 2372632 | SAUSA300_2207 | xanthine/uracil permease family protein | Ala -> Thr | NE283 |
| 2408545 | ureF | urease accessory protein UreF | Met -> Val | NE43 |
| 2412040 | SAUSA300_2247 | staphylococcal accessory regulator | Pro -> Leu | NE210 |
| 2417031 | nhaC | Na+/H+ antiporter NhaC | Val -> Leu | NE1470 |
| 2422550 | SAUSA300_2255 | monooxygenase family protein | Ala -> Glu | NE691 |
| 2435918 | SAUSA300_2265 | putative amino acid permease | Lys -> Ile | NE1435 |
| **2503353** | **rsp** | **transcription regulatory protein** | **Val -> Ala** | **NE1304** |
| 2551471 | SAUSA300_2374 | ABC transporter, ATP-binding/permease protein | Leu -> Phe | NE139 |
| 2578843 | SAUSA300_2395 | amino acid permease | Gly -> Asp | NE1131 |
| 2673918 | ptsG | phosphotransferase system, glucose-specific IIABC component | Pro -> Leu | NE39 |
| 2690087 | SAUSA300_2489 | antibiotic transport-associated protein-like protein | Lys -> Glu | NE1170 |
| 2714644 | SAUSA300_2508 | conserved hypothetical protein | Thr -> Ile | NE1547 |
| 2730990 | SAUSA300_2529 | conserved hypothetical protein | Val -> Ile | NE1042 |
| 2734821 | panC | pantoate--beta-alanine ligase | Pro -> Leu | NE1876 |
| 2755515 | bccT | choline/carnitine/betaine transporter, BCCT family | Phe -> Leu | NE677 |
| 2762932 | SAUSA300_2554 | sulfite reductase flavoprotein | Asp -> Gly | NE238 |
| 2816792 | SAUSA300_2589 | LPXTG-motif cell wall surface anchor family protein | Thr -> Met | NE33 |
| 2834796 | hisF | imidazole glycerol phosphate synthase subunit hisF | Glu -> Gly | NE1232 |
