# Supplementary material for: Evolutionary Trade-Offs Underlie the Multi-faceted Virulence of Staphylococcus aureus
Source: PLoS Biol. 2015 Sep 2;13(9):e1002229. doi: 10.1371/journal.pbio.1002229 (PMC4558032; doi:10.1371/journal.pbio.1002229)
Supplement: S3 Table — (DOCX) [file pbio.1002229.s013.docx]

| **Isolate name** | **Suspected portal of entry** | **Persistent/resolved Bacteraemia**  **(days culture positive)** |
| --- | --- | --- |
| MR005 | Pustule right ankle | Persistent (11) |
| MR007 | TDC infection | Persistent (6) |
| MR018 | Breast abscess | Persistent ((8) |
| MR019 | Septic phlebitis | Persistent (4) |
| MR021 | Central line | Persistent (3) |
| MR022 | Skin abscess | Resolved (n/a) |
| MR023 | Unknown | Resolved (n/a) |
| MR025 | Buttock abscess | Persistent (5) |
| MR026 | Pneumonia | Resolved (n/a) |
| MR027 | Toe ulcer and osteomyelitis | Resolved (n/a) |
| MR029 | Unknown | No information available |
| MR030 | THA joint infection | Resolved (n/a) |
| MR031 | Recrudescence of prior endocarditis | Persistent (5) |
| MR035 | Urinary tract infection | Resolved (n/a) |
| MR036 | Unclear (but had unstageable heel and other LE ulcers, pneumonia on CT) | Resolved (n/a) |
| MR039 | Foot ulcer | Persistent (7) |
| MR047 | Right toe gangrene/cellulitis | Resolved (n/a) |
| MR051 | Left toe osteomyelitis | Persistent (2) |
| MR060 | Pneumonia | No information available |
| MR063 | Pneumonia | Died before repeat sampling |
| MR064 | Severe atopic dermatitis | Resolved (n/a) |
| MR065 | Superinfected LUE graft | Persistent (9) |
| MR072 | Right arm abscess at prior PICC site | Resolved (n/a) |
| MR073 | Cellulitis/Abscess | No information available |
| MR074 | Unknown | N/A first sample post mortem |
| MR077 | Dialysis catheter | Persistent (4) |
| MR078 | Pneumonia | Persistent (4) |
| MR081 | Cellulitis | Resolved (n/a) |
| MR083 | Unclear (+sacral decub, pneumonia, trochanteric fracture after fall) | Resolved (n/a) |
| MR084 | Cellulitis, abscess, osteomyelitis (left tibia) | Persistent (3) |
| MR087 | Purulent cellulitis at IV site | Resolved (n/a) |
| MR090 | Surgical site | Resolved (n/a) |
| MR091 | Right toe osteomyelitis/cellulitis | Resolved (n/a) |
| MR096 | SSTI (face and thigh abscess) | Persistent (2) |
| MR107 | TDC/permanent catheter | Persistent (15) |
| MR110 | Unknown | Resolved (n/a) |
